# Reproducing Polychronization: A Guide to Maximizing the Reproducibility of Spiking Network Models

**DOI:** 10.3389/fninf.2018.00046

**Published:** 2018-08-03

**Authors:** Robin Pauli, Philipp Weidel, Susanne Kunkel, Abigail Morrison

**Affiliations:** ^1^Institute of Neuroscience and Medicine (INM-6) and Institute for Advanced Simulation (IAS-6) and JARA BRAIN Institute I, Jülich Research Centre, Jülich, Germany; ^2^Faculty of Science and Technology, Norwegian University of Life Sciences, Ås, Norway; ^3^Department of Computational Science and Technology, School of Computer Science and Communication, KTH Royal Institute of Technology, Stockholm, Sweden; ^4^Institute of Cognitive Neuroscience, Faculty of Psychology, Ruhr-University Bochum, Bochum, Germany

**Keywords:** reproducibility, polychronization, spiking network models, spike-timing dependent plasticity, synchrony

## Abstract

Any modeler who has attempted to reproduce a spiking neural network model from its description in a paper has discovered what a painful endeavor this is. Even when all parameters appear to have been specified, which is rare, typically the initial attempt to reproduce the network does not yield results that are recognizably akin to those in the original publication. Causes include inaccurately reported or hidden parameters (e.g., wrong unit or the existence of an initialization distribution), differences in implementation of model dynamics, and ambiguities in the text description of the network experiment. The very fact that adequate reproduction often cannot be achieved until a series of such causes have been tracked down and resolved is in itself disconcerting, as it reveals unreported model dependencies on specific implementation choices that either were not clear to the original authors, or that they chose not to disclose. In either case, such dependencies diminish the credibility of the model's claims about the behavior of the target system. To demonstrate these issues, we provide a worked example of reproducing a seminal study for which, unusually, source code was provided at time of publication. Despite this seemingly optimal starting position, reproducing the results was time consuming and frustrating. Further examination of the correctly reproduced model reveals that it is highly sensitive to implementation choices such as the realization of background noise, the integration timestep, and the thresholding parameter of the analysis algorithm. From this process, we derive a guideline of best practices that would substantially reduce the investment in reproducing neural network studies, whilst simultaneously increasing their scientific quality. We propose that this guideline can be used by authors and reviewers to assess and improve the reproducibility of future network models.

## 1. Introduction

Reproducing computational models of networks of spiking point neurons seems like it should be easy. Neuron and synapse models are described as systems of ordinary differential equations with a few additional conditions and constraints. By specifying the parameters, the initial conditions, and any stimulus to the network, the dynamics of any reproduced network should be at least statistically equivalent, or even identical if external sources of random numbers are handled appropriately.

However, this optimistic attitude rarely survives the experience of trying to reproduce a model from a paper. As contributors to the NEST simulator (Gewaltig and Diesmann, 2007), the authors have reproduced a variety of models to create examples. A major source of frustration is inadequate specification of numbers. Parameters are sometimes missing from the description in the paper. This can be in an overt manner, e.g., τ_m_ occurs in the neuron model equations but its value is not stated anywhere, or occur covertly, such that the parameter is not even mentioned in the text. Another common issue with parameters is that the value used in the paper is not the value used for the simulation. Sometimes the number is rounded to a smaller number of decimal places, sometimes it is plain wrong, sometimes the unit is wrong, and sometimes the author fails to mention, for example, a multiplicative factor. Similarly, initial conditions can be incompletely or incorrectly specified, for example the authors state that the initial values for a given parameter are drawn from a certain random distribution, but fail to mention it is truncated.

A further area of divergence is inadequate specification of implementation. One example of this would be for the truncated distribution mentioned above, the authors do not state the behavior when a number is drawn outside of the bounds: clip to bounds or re-draw? Other examples include the choice of the numeric solver of model dynamics and issues to do with event ordering in plastic synapse models – if a pre- and post-synaptic spike arrive simultaneously at a synapse implementing spike-timing dependent plasticity, which happens first, depression or facilitation?

It is worth noting that the two types of insufficient specification are of quite different natures and cannot necessarily be addressed by the same approach. For the majority of current spiking point neuron models, the number of parameters to be specified is large but not ridiculously so. Thus it is reasonable to expect that they all be mentioned explicitly in the main text of a manuscript or in its Supplementary Material. This issue was partially addressed by Nordlie et al. (2009), who developed a break-down of network models into components (e.g., neuron model, connectivity, stimulus etc.) which can then be expressed in tables with a standardized layout. The experience of the authors is that the exercise of filling out these tables brings parameters to light that might otherwise have been overlooked, however it does not provide any protection against wrong values or secret multiplicative factors as discussed above.

In contrast, a complete specification of the implementation cannot be sensibly captured in tables, as it is “how” rather than “what” information. Whereas some aspects can be explained in the text of a manuscript, comprehensive coverage cannot be expected, firstly because it would make manuscripts technically dense to the point of unreadability, and secondly because human readable language is rife with ambiguities that would hamper an accurate reproduction of the described model. Because of these specification issues it is often not possible to reproduce a model from a paper without contacting the authors and extracting more information.

Clearly, then, sharing model code should be seen as part of a modeler's obligation to enable reproducibility of his or her study. This is easily achieved on a variety of platforms. However, downloading a model code from such a platform and running it on your own machine does not constitute reproducing a study in the strong sense. Using the definitions proposed by the Association for Computing Machinery (2016), we will refer to this as *replication*, i.e., different team, same experimental set-up (see Plesser, 2018 for a summary and analysis of different technical definitions for reproduction and replication). At best, it simply shows that the model code is portable and generates the reported results. At worst, it does nothing, since availability of code does not entail that this code can be run on your machine, as tragically documented by Topalidou et al. (2015) and on a more industrial scale (but outside the neuroscience context) by Collberg and Proebsting (2016).

The ReScience Initiative (Rougier et al., 2017) seeks to address this issue by providing a home for *reproductions* of model studies (i.e., different team, different experimental set-up). The reproductions published there are open-source implementations of published research that are tested, commented, and reviewed. However, it would be preferable if the original publications were intrinsically reproducible, rather than requiring intense post-publication efforts by others. To achieve this, it is important not only for researchers to put greater effort into making their code available and comprehensible, but also for reviewers to be able to quickly evaluate any factors that might undermine its reproducibility.

In this article, we develop a guideline for spiking neuronal network modelers to present their work in such a way as to minimize the effort of other scientists to reproduce it. As we believe that concrete illustrations are necessary to convincingly motivate recommendations, we provide these by reproducing a seminal study in computational neuroscience, a minimal network generating polychronous groups (Izhikevich, 2006). We analyze which features of the model and analysis code (and description in the manuscript) support, and which hinder, the reproduction of the study. From each of these features, we derive a corresponding recommendation that, if followed by future studies, would increase their reproducibility.

The choice of this source material is motivated by the following considerations. Firstly, the author took the (then) highly unusual step of making the model code available, both in the manuscript itself (MATLAB) and in downloadable form (MATLAB and C++). Secondly, despite the availability of the code, the model is rather challenging to reproduce, due to a number of non-standard models and numerics choices, thus making it a fruitful source of recommendations.

We would like to emphasize that the choice is purely demonstrative, and almost any study with published code could have been used for our purposes; indeed the authors' own work has not come up to the standards we propose. Furthermore, we point out that many of the technical solutions we propose were not available at the time the source material was published.

In the first phase (Section 2.2), we establish a baseline by downloading the author's C++ code and carrying out some minimal modifications to enable it to run locally. In the second phase we demonstrate that our best-effort initial attempt to reproduce the study's results using a NEST implementation of the network fail (Section 3.2), and focus our efforts on creating an implementation capable of reproducing results identical on the level of individual spikes and synaptic weights. For this implementation, various artifacts (e.g., connectivity matrix) need to be exported from the original implementation into NEST. Therefore, in the the third phase we develop a stand-alone NEST implementation and investigate how well it reproduces the original results (Section 3.3). We demonstrate that the original network has a second activity mode unreported in the original study.

In Section 3.4, we manipulate the stand-alone NEST implementation to investigate various issues with respect to numerics and model features that we discovered in the preceding phases, and in Section 3.5 we perform an analogous investigation of the main analysis algorithm provided as part of the original code. In this way, we uncover unreported major dependencies on coding errors and implementation (rather than conceptual) choices such as the background noise, the resolution of the neuron update and the thresholding parameter of the analysis algorithm. The series of recommendations that we derive from reproducing the original model and investigating its sensitivity to parameters and implementation details are gathered and discussed in Section 3.6.

Our results demonstrate that putting effort into code presentation and study design to boost its reproducibility does not just make it easier for future researchers to independently confirm the results and/or extend the model. It also increases the scientific quality of the study, by reducing the risk that results have been distorted by avoidable coding errors, inappropriate choices of numerics, or highly specific parameter settings.

## 2. Methods

Our implementation of the model and all materials used for this study are publicly available on GitHub^1^ under MIT license.

### 2.1. Polychronization network model

The polychronization network model as described in Izhikevich (2006) is inspired by a patch of cortical tissue. The network model contains 1, 000 neurons, of which 800 are modeled as excitatory and 200 modeled as inhibitory, as described in Izhikevich (2004). Throughout the simulation the neurons are stimulated by the unusual method of randomly selecting one neuron in each millisecond step, and applying a direct current of 20pA to it for the duration of that step, reliably evoking a spike.

The neurons in the original network model are connected as follows: for each inhibitory neuron, 100 targets are selected from the excitatory population, not permitting multapses or autapses. Inhibitory synaptic connections are static with a weight of −5mV and argued to be local, and thus have a delay of 1ms. For the excitatory neurons, the 100 targets are selected at random from the whole network, also not permitting multapses or autapses. Excitatory synaptic connections are plastic, the detailed dynamics of which are described in Section 3.4.2. The delays for these connections are highly structured: they are evenly spread between 1 and 20ms, i.e., exactly five outgoing connections of each neuron have the delay 1ms, exactly five connections have the delay 2ms, and so on. The model parameters are summarized in tables in the Supplementary Materials.

### 2.2. Preparing the polychronization network model for replication

We downloaded the C++ source code poly_spnet.cpp from the author's website^2^ and installed it locally. The original code could not be compiled with the standard g++ compiler under Ubuntu 16.04 LTS. Some minor adjustments were necessary to make the code compile and run, which are given in the Supplementary Material. We note that there are differences between the MATLAB code published in Izhikevich (2006) and that available for download, and between both MATLAB versions and the C++ code. Unless stated otherwise, all remarks on features of “the original code” refer to the C++ version used as the basis of this study.

The source code is a single standalone script that comprises both simulation and the analysis, including identification of polychronous groups. In order to later compare the statistics of groups found by the original simulation code and by the NEST re-implementations, we re-structured the code to separate the analysis from the simulation, writing the neural activity (spike times and membrane potentials) in NEST-formatted text files, and the network connectivity in JSON format.

We checked that using the separated versions of the simulation script and analysis script serially yields identical results to running the downloaded code with integrated simulation and analysis. This enabled us to run the same analysis on data produced by the original code and by implementations in NEST, rather than having to chase down disparities in simulation and analysis code simultaneously. In the following, we refer to this slightly modified version of the downloaded code as the “original network model”.

The recommendations that we compile in Section 3.6.1 are mostly inspired by this initial process of assessing and adapting the original source code.

#### 2.2.1. NEST

Network simulations are either carried out using Izhikevich's homebrewed network simulator written in C++ or using NEST 2.14. (Peyser et al., 2017). The source code for the Izhikevich synapse model is publicly available on GitHub in a branch of a fork of the NEST repository. Due to the model's multiple idiosyncrasies and numerical issues reported on in this article (see Section 3.2.2 and Section 3.4), it does not fulfill the quality standards for NEST and so will not be merged to the master branch of the main repository and included with future releases of NEST.

### 2.3. Experiments

#### 2.3.1. YAML

In order to investigate the dynamics of the model under study, we defined several experiments described in Section 3.4. In the experiments we varied parameters such as stimuli, delay distributions and numeric resolution. For each experiment we wrote a distinct parameter file in YAML (“Yet Another Markup Language” or “YAML Ain't Markup Language”) which makes the workflow very clear and modular. All YAML files are available in the repository and in the Supplemental Material in tabular form.

#### 2.3.2. Polychronous group finding algorithm

For our data analysis we used two different versions of the algorithm which finds polychronous groups. For the first version, we extracted the original algorithm from Izhikevich's C++ code and adapted it such that it uses our JSON data format. We confirmed that our adaptation does not change the original results by comparing the found groups on a given dataset.

This C++ version of the algorithm finds groups by running a full network simulation, in which a specific group of neurons is stimulated and the network response recorded. As the delay distribution is hardcoded in the structure of the algorithm and the integration timestep is fixed to 1ms, it is not possible to apply this algorithm to experiments in which we changed these parameters. We therefore wrote a second algorithm in Python that runs a NEST simulation, in which we can easily alter the integration timestep or delay distribution.

For the Python version of the algorithm we tried to be as close as possible to the original C++ version, but generalized to be applicable to all parameter sets. Starting from a pivot neuron, we iterate over all triplets of neurons (“anchor neurons”) forming synapses with at least 95% of the maximal weight to this pivot neuron. We then determine all other neurons which are targeted by this triplet, start a NEST simulation, and stimulate the targeted neurons in the order of their delay relative to the pivot neuron, with the corresponding weight from the triplet. We record the network response and consider the triplet and all neurons emitting a spike during the simulation as part of a polychronous group. After the NEST simulation finishes we scan the connectivity for connections between the neurons participating in this group and define the “layer of a neuron” as the length of the chain of pre-synaptic neurons within the group. Finally, following the original algorithm, the group is only considered to be relevant if the longest path is larger than seven layers and all three anchor neurons participate in the activation of the group. We point out that setting the minimum layer threshold lower than seven leads to a rapid increase of the number of groups. The Python version deviates from the original C++ code, as we found errors in the original code that we fixed in our version. For example, for large groups, the original code exhibits an array index overflow, leading to erroneous spike delivery during group detection. Moreover, the original code often misses one last spike in the network response; this reduces the group size and longest path by one, leading to a reduced number of relevant groups.

Compared to the original algorithm, our Python version typically finds twice as many polychronous groups. However, conceptually the two algorithms seem to be approximately equivalent as nearly all (>99%) groups found by the original version are also found by the Python version. Thus, we consider the Python version to be a “generous” evaluation of the number of groups with respect to the original version. A detailed discussion of the group finding algorithm and the definition of polychrony can be found in Section 3.5.

#### 2.3.3. Activity metrics

To estimate firing rates, we binned the spikes of all excitatory (inhibitory) neurons in bins of 5 ms. We then divided by the number of excitatory (inhibitory) neurons to calculate the average rate of one neuron in the population *f*_pop_ in spks/s. The single neuron variability is expressed by the coefficient of variation (CV) of the inter-spike interval distribution, *CV* = σ(*ISI*)/μ(*ISI*). The synchrony of the network dynamics is calculated as the Fano factor (FF) of the population averaged spike counts *N*(*t*) with FF = σ(*N*(*t*))^2^/μ(*N*(*t*)). To estimate the spectral characteristic of the network, we applied a Fourier transformation on the population rate *f*_pop_ of the excitatory neurons, following Izhikevich (2006). We calculated the peak frequency in the range between 20−500Hz and categorized the network activity as having a low gamma peak if its maximum amplitude fell in the range 35−50Hz, and a high gamma peak if the maximum amplitude fell in the range 50−100Hz.

#### 2.3.4. Snakemake

**Figure 1:**
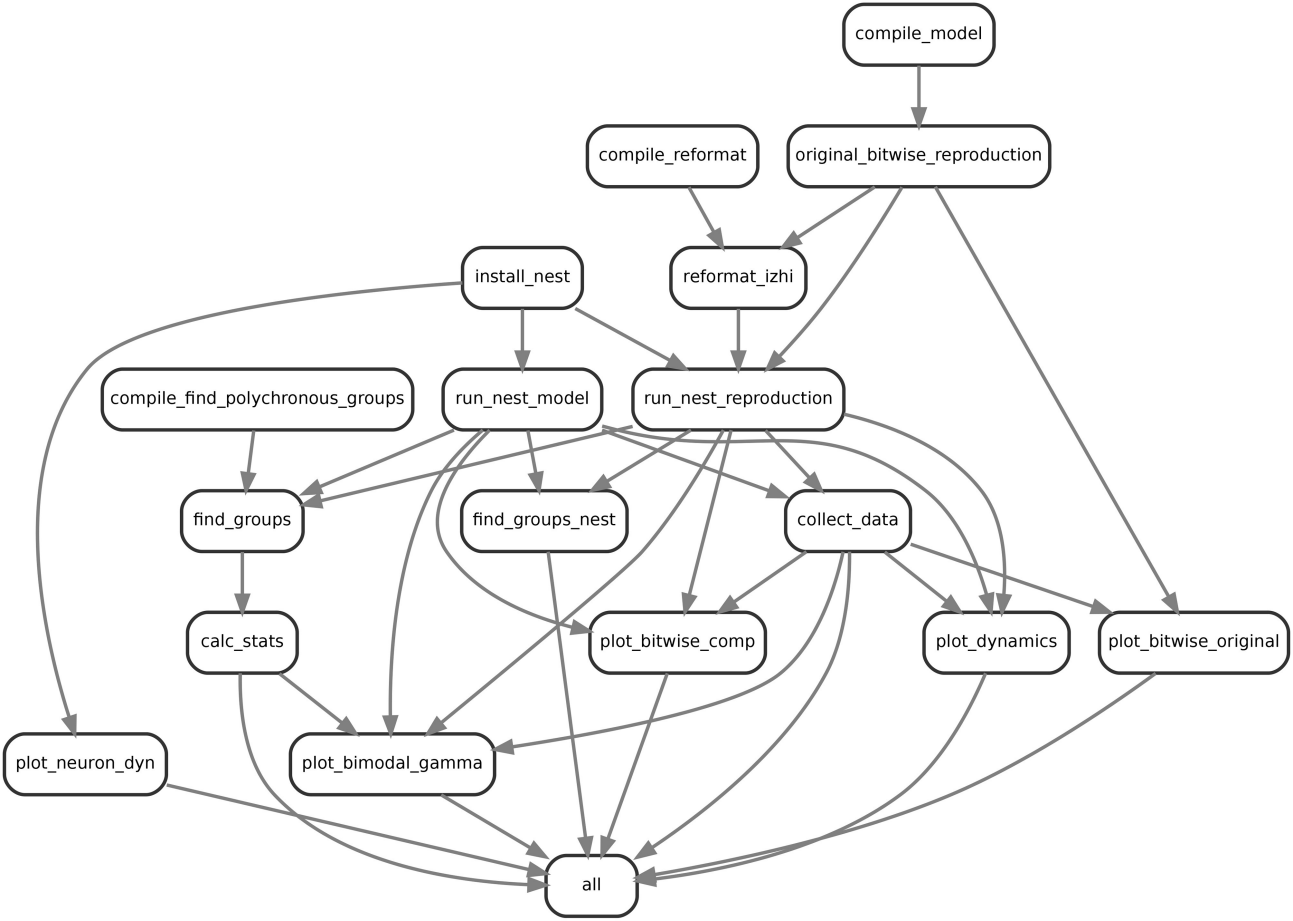
Example visualization of the snakemake workflow for comparing the bitwise reproduction and the qualitative model. Shown are the rulenames defined and their input output relationships.

Snakemake is a script based workflow management system which allows reproducible and scalable data analysis (Köster and Rahmann, 2012). The complexity of our simulations, involving several different versions of neuron, synapse and network models as well as analysis scripts was massively eased by using snakemake. It allows its users to run their analysis on laptops and clusters, visualize the workflow (see Figure 1) and manage the data in a consistent and efficient way. Using a workflow manager enables us to keep track of the files generated by the original code, the slightly modified version of the original code and the various experiments conducted in sections 3.4 and 3.5. Snakemake links the version of the program to the data it created, such that it can re-run specific sections of a workflow depending on what parts were changed.

### 2.4. Workflow

In order to investigate the dynamics and performance of the model under study on different sets of parameters (see our recommendations in Section 3.6.3), we simulated the model many times under different conditions which led to a rather complex workflow. This is illustrated in Figure 1 for the example of comparing the bitwise reproduction to the qualitative reproduction. Shown are the rulenames (labels in the boxes) defined and their input-output (arrow between boxes) relationships, for example *collect_data* where the arrows indicate that the defined rule uses input, i.e., a data file from *run_nest_model* and its output is used by *plot_dynamics*.

First, to prepare the simulations we have to compile and install all dependencies including the original model in C++ (*compile_model*), the tools for reformatting the original data to JSON (*compile_reformat*), the original algorithm to find polychronous groups (*compile_find_polychronous_groups*) and NEST (*install_nest*). Next, we run the original model (*original_bitwise_reproduction*) and reformat the produced data to use the JSON dataformat (*reformat_izhi*). The output of the original model is used to initialize the neurons, connectivity and stimuli in the NEST bitwise reproduction. We run the bitwise reproduction (*run_nest_reproduction*) and the qualitative reproduction (*run_nest_model*), which is independent of the output of the original model. Afterwards we collect all data (*collect_data*) and run the algorithm for finding polychronous groups (*find_groups* and *find_groups_nest*). Finally we calculate group statistics (*calc_stats*) and activity statistics and plot the relevant data (*plot_dynamics*). The box with label *all* is a dummy target used to define all files that should be produced by the workflow. This is used by Snakemake to generate the dependency tree in Figure 1. This workflow is repeated automatically 10 times for all experiments and 100 times for bitwise reproduction and qualitative reproduction using Snakemake. After generation of the necessary files, the plots for the single neuron dynamics (**Figure 9**), group analysis (**Figure 8**) and the bi-modal dynamic states (**Figures 5, 7**) are produced.

## 3. Results

### 3.1. Replicating the polychronization network model

The polychronization network model was proposed by Izhikevich (2006) as a minimal spiking neural network model capable of exhibiting polychronization, consisting of randomly coupled point neurons expressing STDP (see Section 2.1 for a detailed network description). In addition to the network model, an algorithm for detecting polychronous groups was provided in this study. A polychronous group is a group of neurons connected in such a way that neural activity propagates in a reliable and stereotypical fashion due to the interplay between synaptic delays and the activation times of neurons. Izhikevich (2006) illustrates the concept of polychrony in a comprehensible way and links to higher neural processes such as cognition, computation, attention and consciousness.

**Figure 2:**
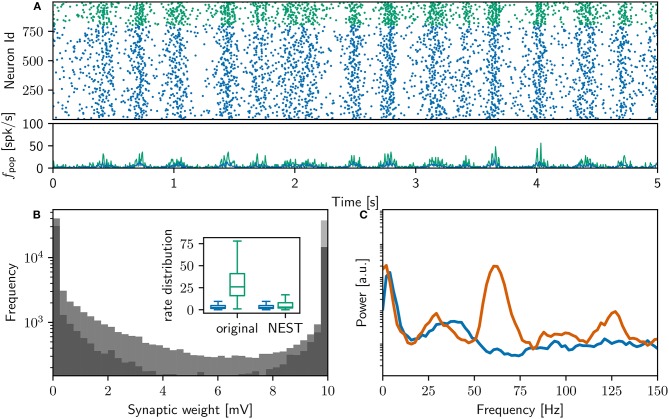
Comparison of initial NEST network model with original. **(A)** Spike raster plot and rate envelope generated by the NEST simulation in the final 10 s (17, 990−18, 000) for inhibitory (green) and excitatory (blue) neurons. **(B)** Final weight distribution (frequency plotted on a logarithmic scale) for the original (dark gray) and NEST (light gray) simulations. Inset: rate distributions over the final 10 s displayed as box plots for the excitatory and inhibitory populations in the original and NEST simulations, colors as above. **(C)** Power spectrum of the rate envelope over the final 10 s for the excitatory population in the original (orange curve) and NEST (blue curve) simulations.

Our execution of the original network model, prepared for execution on our system as described in Section 2.2, successfully replicates the main results reported in that study. Executing the original network model on our system results in 18,000 s of network activity, exhibiting slow oscillations and gamma rhythms (interpreted by the original study as “sleep-like” and “implicated in cognitive tasks”). After simulation, the original polychronous group finding algorithm (see Section 2.3.2) identifies 4, 305 polychronous groups in the connectivity of the network model. The final weight distribution and power spectrum can be seen in Figure 2.

### 3.2. Identical reproduction

In order to reproduce a network model in machine precision it is not enough to parameterize the network model identically. The issue of reproducibility goes deeper than the model specification itself. For example the choice of compiler, the order in which numerical operations are executed or the underlying hardware the model is run on can lead to rounding errors; these accumulate over a long simulation time and therefore lead to different results. Without providing the original code with provenance tracking and raw data, a model can therefore not be reproduced identically as there is no possibility to compare the exact results, i.e., every spike and every weight (Ghosh et al., 2017).

The original raw data was not provided, but given that the original code is written in C++, as is NEST, we determined that it should be possible to replicate the results yielded by the original C++ version on our machines with a NEST version on the same machine. This is not what is normally understood as “reproduction of a neural network model,” which would typically only aim for statistical equivalence of aggregate findings, e.g., firing rates, mean number of groups, etc. For such measures, environmental features such as the operating system or compiler version should not play a role; if they do, this suggests the model is inherently excessively sensitive. However, we take this step here to ensure we have captured all details of the neuron and synapse model used in the original network model.

#### 3.2.1. Initial iteration

The Izhikevich neuron model (Izhikevich, 2004) used in the original code already existed in the NEST code base, but we needed to implement the synapse model based on the text description in Izhikevich (2006) and the modified version of source code as described in Section 2.2.

The original network model has three sources of randomness: the selection of which neurons to connect, the initial values of the membrane potentials, and the noise stimulation, implemented as a direct current delivered to one randomly selected neuron in each millisecond. We therefore modified the original version to save the connectivity matrix, the initial values of the membrane potentials and the order of neuron stimulation to file; these are then read in and applied by the NEST simulation. Additionally, we modified the original to allow the seed for the random number generator to be set as a parameter, thus enabling multiple runs of the model to be carried out in our snakemake workflow (see Section 2.4).

Figure 2A shows a raster plot of the spiking activity in the final 10 s of simulation in our initial iteration of trying to replicate the original model identically; the rate distributions are strikingly different, in particular the inhibitory rate of the NEST model is low compared with the original version (see Figure 2B, inset). The power spectra (Figure 2C) reveal that the strong gamma peak exhibited by the original model is not present in the NEST simulation. The weight distribution is also different; whilst still maintaining a bimodal character, the NEST simulation has a larger number of maximum weights (Figure 2B). Using the original algorithm to find polychronous groups we were able to find five groups in ten iterations with different random seeds.

These results show that despite the best, good-faith attempt of a group of researchers with considerable experience in developing neuron, synapse and network models, it was not possible to reproduce the neuron and synapse dynamics described in Izhikevich (2006) in one pass, even though the source code was available for inspection. Our first iteration fails to reproduce the key findings of the original study, either in terms of network dynamics or in terms of the generation of polychronous groups. Not only does this demonstrate that reproduction of computational models can be challenging even for experienced modelers with access to the original model code, it also raises the possibility that the aforementioned key findings are dependent on implementation details of the synapse and neuron models.

#### 3.2.2. Final iteration

It took a great investment of time to iteratively adapt the NEST simulation described above such that it yielded identical results to the original version. There were a number of disparities in the respective neuron and synapse models, including priority assigned to simultaneous events in the synapse model, ordering of neuron update, implementation of exponential functions, and ordering of mathematical operations.

These algorithmic and numeric aspects are (understandably) not discussed in the text description of the manuscript, underlining once again the importance of sharing the code. However, neither can they be readily found by examining the C++ code, as it is rather hard to comprehend in detail for the following reasons:

- It is uncommented (or commented only with the corresponding lines from the MATLAB version of the code)
- It exhibits low encapsulation; neuronal and synaptic updates are mixed throughout the simulation code, and synaptic interactions rely on long nested sequences of indexing rather than meaningfully named functions
- Numerics are not always standard, e.g., multiplication by 0.95 in each time step rather than using an exponential function
- Parameters are not always given meaningful names and defined in one place, such as the beginning of the script or in a separate parameter file; moreover, some appear as “magic numbers” in the middle of the code

We note that the MATLAB code is somewhat better commented, but the discrepancies between the sources mean that comments in one do not necessarily help to understand the other. However, even with a perfectly structured and commented source code it would be difficult to find all disparities, as there are many special cases in the particular synaptic plasticity algorithm used in the original network model. It would be very challenging to think of all possible special cases and check by mental simulation of the two codes whether each one would be handled identically.

Consequently, it was necessary to write several specific tests for the neuron and synapse model in both the original version and the NEST implementation in order to progressively eliminate discrepancies until they all came to light (see Section 39 in the recommendations). By comparing their responses to identical input, especially border cases, it was possible to track down the algorithmic differences between the models. In the case of NEST, writing scripts to test a synapse or neuron with a particular input is easy, because it is a modular simulation tool written in a general purpose fashion, i.e., not optimized for a specific network model. In contrast, as the homebrewed original version is neither modular nor general, testing the behavior of individual elements required some creative modifications (see our recommendations in Section 3.6.2).

The main discrepancies between the original version and our initial attempt, which we resolved in the bitwise reproduction, were as follows:

- The STDP spike pairing in the original model is of type nearest-neighbor, whereas the default pairing in NEST is all-to-all (see Morrison et al., 2008 for a review)
- The original neuron model processes incoming spikes at the beginning of a timestep, rather than the end, as in NEST, leading to a shift in delivery times of 1ms and thus overall weaker synapses (see STDP windows of the initial and bitwise reproduction in Figure 4)
- For border cases, e.g., synchronous spiking of pre- and post-synaptic neurons, the original synapse model applies the LTP and LTD in a different order from our initial reproduction
- The original C++ model applies a decaying term to the eligibility trace before adding it to the synaptic weights, whereas our initial attempt (and the MATLAB version) applied it afterwards:
  wdev← wdev^*^0.9;
  weight← weight + 0.01 + wdev;

Note that the C++ and MATLAB versions of the code diverge in their handling of the eligibility trace, and the variables wdev and weight (the buffered weight changes and the current synaptic weight), are known as sd and s in the original code.

These four disparities in the synapse model lead to the largest differences in the two models. However, even after aligning these, we observed that the spike trains of the original and our re-implementation could be identical for several hours of simulation before some small differences in spike timings ultimately led to complete divergence. This was due to some extremely small (i.e., around machine precision) deviations, which were inflated by the instable numerical integration. We therefore had to additionally adjust all numerical operations to be in the same order as in the original code, and reverse any conversions to standard numerics.

**Figure 3:**
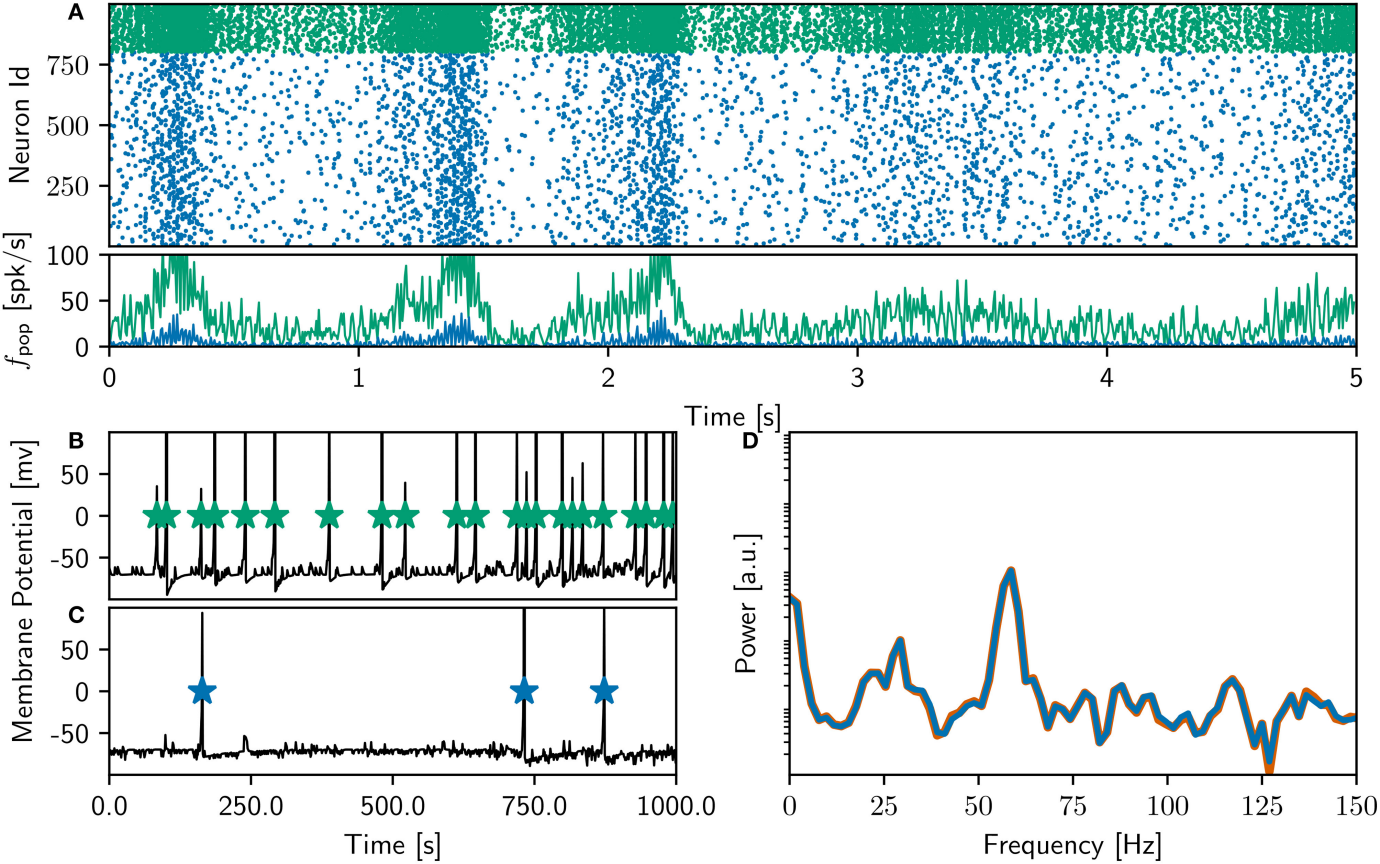
Comparison of bitwise identical NEST network model to original. **(A)** Spike raster plot and rate envelope generated by the NEST simulation in the final 10 s (17, 990−18, 000) for inhibitory (green) and excitatory (blue) neurons. **(B)** membrane potential for a selected inhibitory neuron from the NEST simulation and spike times of corresponding neuron from original code. **(C)** As in **(B)** for a selected excitatory neuron. **(D)** Power spectrum of the rate envelope over the final 10 s for the excitatory population from the NEST (blue curve) and original (orange curve) simulation.

- In the eligibility trace, replace exp(−Δ*t*/20) by 0.95^Δ*t*^
- In the neuron update, replace
  0.04 ^*^ v ^*^ v + 5 ^*^ v + 140. − u + I
  by
  (0.04 ^*^ v + 5) ^*^ v + 140. − u + I
- In the synapse update, replace
  wdev^*^ = 0.9;weight + = 0.01 + wdev;
  by
  weight + = 0.01 + wdev ^*^ 0.9;

Finally, after detailed investigation and adjustments to the NEST implementation of the neuron and synapse model, the NEST simulation yielded identical results to the original version over the entire 18,000 s simulation period. Figure 3 shows a raster plot of the spiking activity in the final 10 s of simulation for a NEST network model that replicates the original model identically. It is unquestionable that if the original study had complied with the recommendations in Section 3.6.2, the process of identically reproducing the results would have been far less complicated.

**Figure 4:**
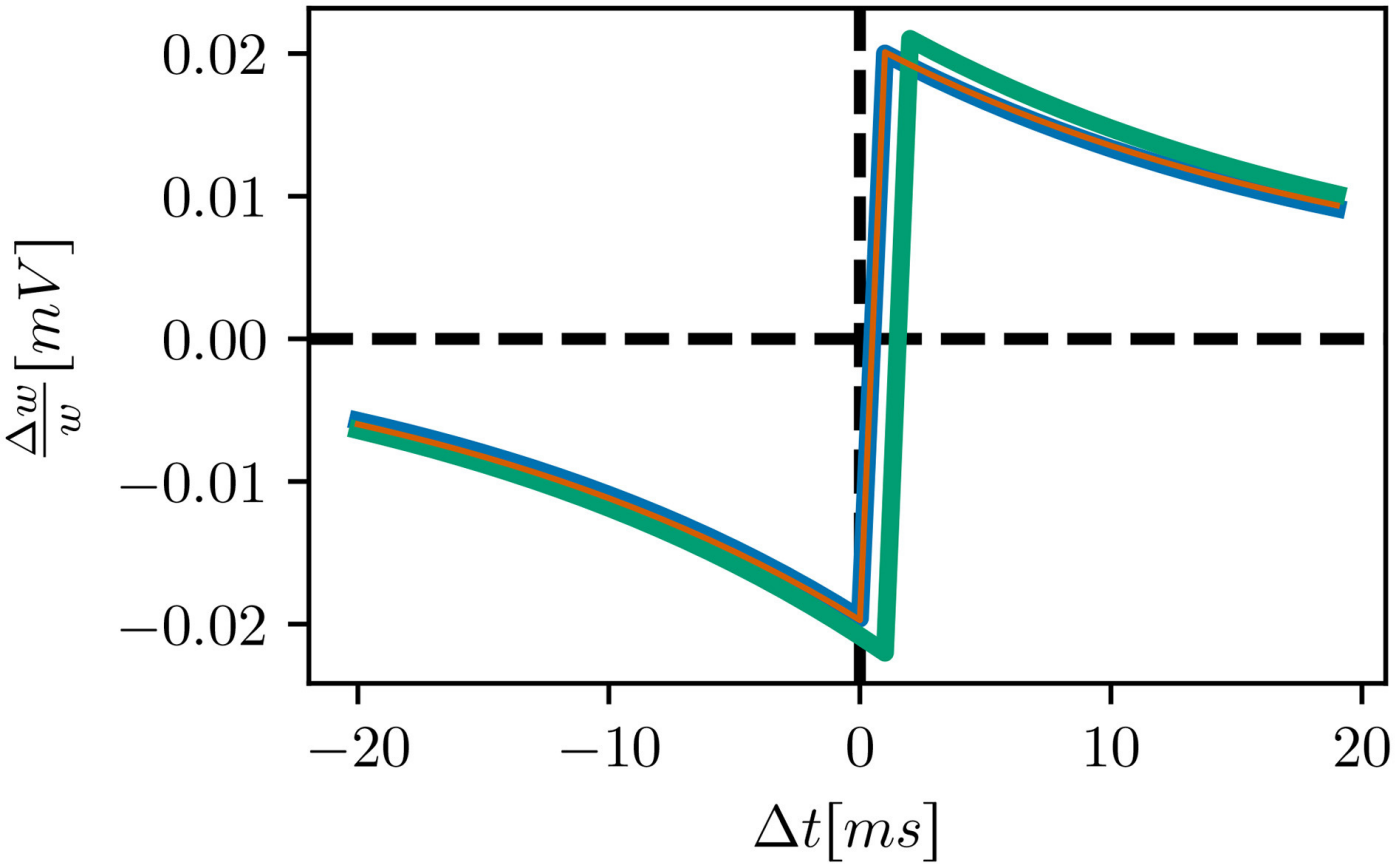
STDP windows of alternative STDP implementations: initial implementation (green), bitwise identical implementation (blue) qualitatively equivalent implementation (orange). Our initial attempt is similar to the bitwise identical reproduction, but shifted by 1ms to positive delays. The windows of the bitwise identical and qualitatively equivalent implementations coincide.

The rate of the inhibitory population is high compared to the NEST network activity shown in Figure 2, and the oscillations in the gamma band are more strongly represented, as can be seen in the power spectrum in the bottom right panel. The bottom left panel demonstrates, for an example inhibitory neuron (top) and an example excitatory neuron (bottom), that the spike times of the NEST simulation coincide with those of the original. The membrane potential in the NEST simulation is recorded after the numeric update step, but before spikes are detected and the membrane potential set back to resting potential. This leads to the membrane potential reaching values of above 100mV, frequently reaching values of around 1, 000mV (**Figure 9**). This indicates numerical instabilities when simulating the neuron model with a resolution of 1ms, which we investigate further in Section 3.4.4.

**Figure 5:**
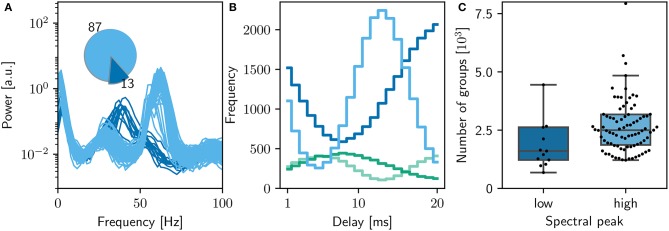
Sensitivity of network dynamics of the bitwise identical NEST implementation to choice of random seed. **(A)** Power spectrum of the rate envelope over the final 10 s for the excitatory population for 100 different seeds. Light blue curves indicate runs resulting in a high gamma peak (60 Hz), dark blue curves those with a low gamma peak (40 Hz). Inset shows the proportion in which these two activity profiles occur. **(B)** The equilibrium distribution of weights (Maximum 10mV, blue curves; minimum 0mV, green curves) as functions of the delay in the high (light) and low (dark) gamma dynamical states. **(C)** Relationship between dynamical state and number of polychronous groups found. Boxes show median and interquartile range (IQR); whiskers show additional 1.5 × IQR or limits of distribution.

The original study showed an analysis of the polychronous groups for exactly one run. To investigate the properties of the distribution of groups, we performed 100 runs of the bitwise identical NEST simulation using different random seeds. Surprisingly, we discovered that the network model does not converge to a single dynamic and structural state, as demonstrated in Figure 5. In the majority of cases (87%), the network activity results in a power spectrum with a high gamma peak around 60 Hz, as previously shown in Figures 2C, 3D. However, the rest of the simulation runs result in a lower peak at 40Hz, an eventuality not reported in the original study. The full collection of power spectra is shown in Figure 5A. The two dynamical states correspond to two alternate structural states. For the high gamma state, the maximum weight of 10mV occurs for delays between 12 and 14ms and for the low gamma state, this maximum occurs for a delay of 20ms (Figure 5B).

Analyzing the polychronous groups (Figure 5C) reveals that the two dynamical/structural states described above develop significantly different numbers of groups. The distribution of numbers of groups detected is shown in Figure 5C. For the high gamma state, the mean number of groups detected was 2, 500 with a inter quartile range (IQR) of 1,300, with a minimum of 1, 200 and a maximum of 23, 000 over the 87 trials resulting in that state. For the 13 low gamma runs, a mean of 1, 600 groups with an IQR of 1,400 were detected (minimum: 700; maximum: 29, 000). Notably, both distributions are lower than the figure of 5, 000−6, 000 reported in the original study.

### 3.3. Qualitative reproduction

The network model developed in Section 3.2 replicates the original results precisely, but this does not coincide with the common understanding of reproducing a model. Firstly, requiring equality of floating-point numbers at machine resolution is too strict, and generally not practicable - here we had the advantage that the original code and the code of the target simulator NEST are both in C++, and so identical sequences of mathematical operations will be compiled into identical machine code. Secondly, all pseudorandom elements need to be extracted from the original code in order to initialize the code used for reproducing the model.

We therefore developed a network model that reproduces the original in the commonly understood sense, i.e., all concepts of the original are faithfully translated into the new framework. Specifically, the sources of randomness (connectivity, membrane potential initialization and neuron stimulation) are replaced with analogous routines within the NEST simulation script, and hence there is no dependence on output from the original model. Moreover, the numerics of the synapse model comply with standard forms, and the simulation is parallelized for multithreaded execution.

It could be argued that such a qualitative reproduction should also integrate the neuron dynamics at a finer resolution than the 1 ms used in the original version, as the resolution of a simulation or the algorithm chosen to numerically solve the dynamics should not be considered a conceptual element of a model. However, it turns out that the numerical integration of the dynamics is critical for the model behavior, which we examine in greater detail in Section 3.4. We therefore remained with the original numerical choices to create the qualitative reproduction of the model.

**Figure 6:**
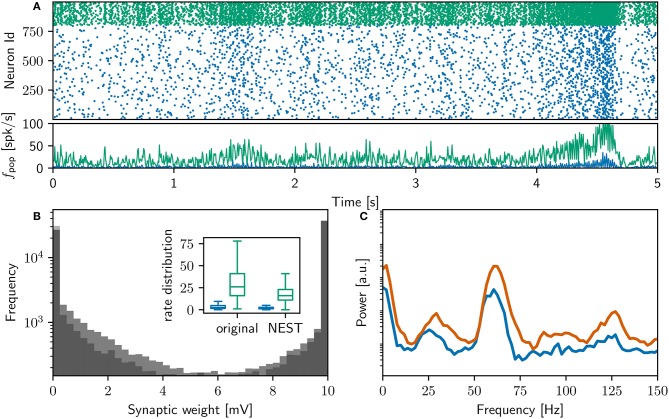
Comparison of qualitatively equivalent NEST network model to original. **(A)** Spike raster plot and rate envelope generated by the NEST simulation in the final 10 s (17, 990−18, 000) for inhibitory (green) and excitatory (blue) neurons. **(B)** Final weight distribution (frequency plotted on a logarithmic scale) for the original (light gray) and NEST (dark gray) simulations. Inset: rate distributions over the final 10 s displayed as box plots for the excitatory and inhibitory populations in the original and NEST simulations, colors as above. **(C)** Power spectrum of the rate envelope over the final 10 s for the excitatory population in the original (orange curve) and NEST (blue curve) simulations, colors as above.

**Figure 7:**
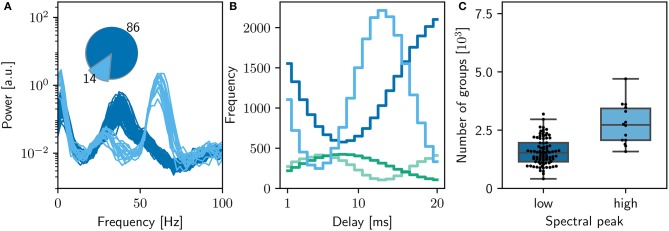
Sensitivity of network dynamics of the qualitatively equivalent NEST implementation to choice of random seed. **(A)** Power spectrum of the rate envelope over the final 10 s for the excitatory population for 100 different seeds. Light blue curves indicate runs resulting in a high gamma peak (60Hz), dark blue curves those with a low gamma peak (40Hz). Inset shows the proportion in which these two activity profiles occur. **(B)** The equilibrium distribution of weights (Maximum 10mV, blue curves; minimum 0mV, green curves) as functions of the delay in the high (light) and low (dark) gamma dynamical states. **(C)** Relationship between dynamical state and number of polychronous groups found. Boxes show median and interquartile range (IQR); whiskers show additional 1.5 × IQR or limits of distribution.

Figures 6, 7 demonstrates that the qualitative reproduction captures the key features of the original model. The raster plots are visually similar to those shown in Figure 3A, an impression supported by the similarity of the rate distributions (Figure 6B, inset) and the power spectra (Figure 6C) to those of the original model. Likewise, the final weight distributions (Figure 6B) overlap almost completely. In line with the bitwise reproduction, simulations exhibiting a high gamma peak yield more groups than the simulations exhibiting a low gamma peak (median 2, 700, IQR 1,300 vs. median 1, 500 IQR: 800; Figure 7C).

However, despite the apparent good match between the qualitative reproduction and the original, analyzing the activity from 100 simulation runs with different random seeds reveals that the proportion of high gamma and low gamma states have reversed (14 high gamma simulations, 86 low gamma simulations) with respect to the bitwise identical reproduction (compare Figure 5A and Figure 7A).

A full investigation of the mechanism by which the network converges to one dynamic state or the other, and the implementational differences between the bitwise identical and qualitatively equivalent NEST simulations that cause a differentiation in the respective likelihoods of these states, lies outside the scope of the current manuscript. However, this result does highlight the importance of the recommendation made in Section 3.6.3: performing multiple runs so that one can discover, and report, alternate dynamical states for a network model. A researcher may have implemented everything correctly, and yet still fail to reproduce key results, if he or she was unlucky enough to select a random seed that caused the network to converge to an unreported, but completely valid, dynamical regime.

### 3.4. Generalizing reproduction

Creating a scientific model by necessity requires making simplifying assumptions. In order to draw credible conclusions on how the brain works from the results of simulating a simplified model, it is therefore important to be vigilant that it is not precisely those simplifying assumptions that cause the reported phenomena. Moreover, when a mathematical model is implemented in code for simulation, this introduces the risk that the numerical approach chosen is not suitable to evaluate the model dynamics with adequate accuracy. If the numerics are not suitable, the reported phenomena may be contaminated with misleading numerical artifacts. Even if the simplifying assumptions are valid, and the numerics well-chosen, the selection of parameters may give results that are a special case, and not representative either of the model or of the targeted physical system.

**Figure 8:**
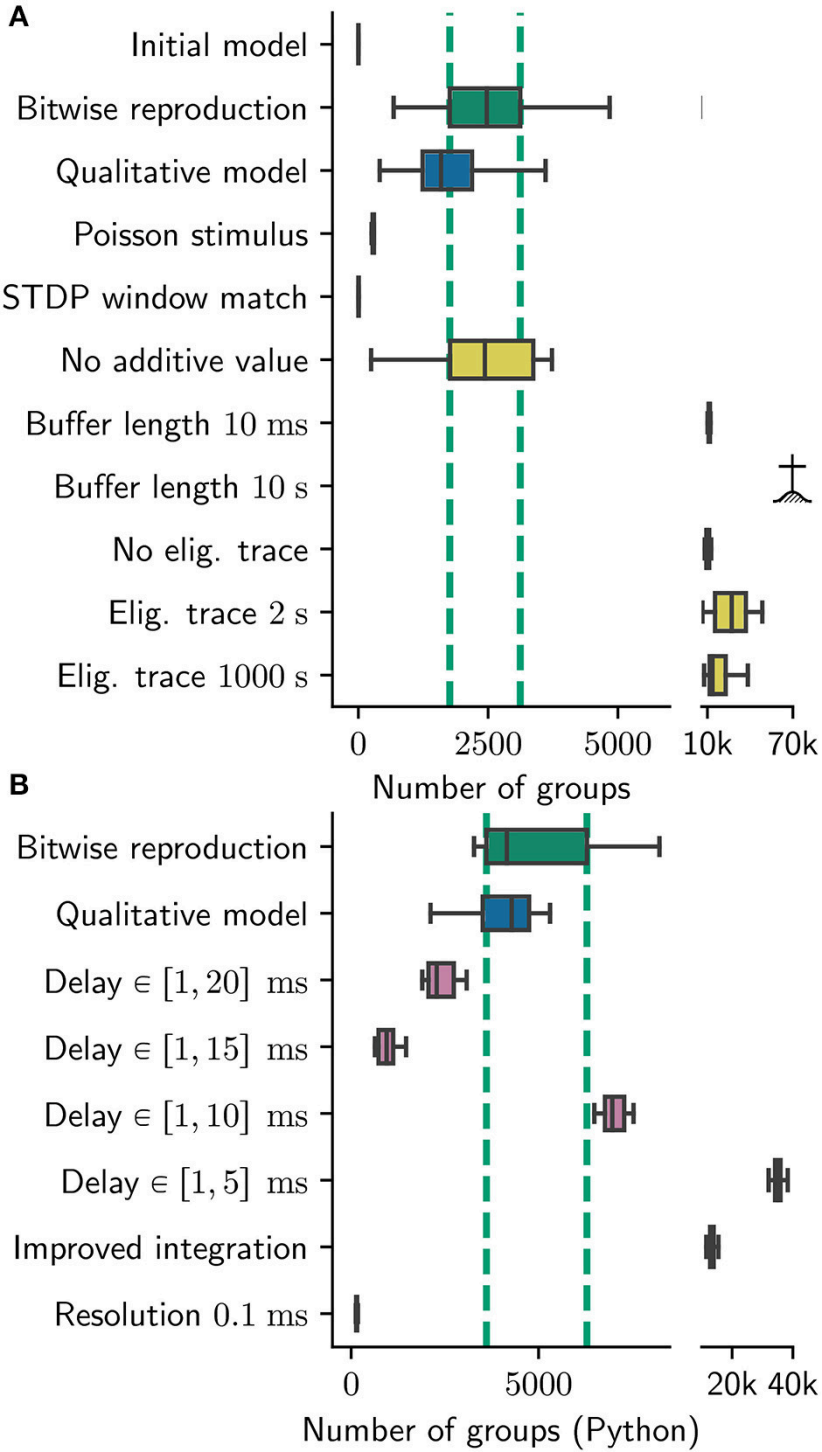
Sensitivity of number of groups found to various parameters. Number of groups found for **(A)** the original group finding algorithm, **(B)** the Python group finding algorithm. Note the different scales; the Python algorithm find about twice as many groups (see Section 2.3.2). Boxes show median and interquartile range (IQR); whiskers show additional 1.5 × IQR or limits of distribution. Group statistics are measured over 100 realizations in the case for bitwise reproduction and qualitative model in **(A)** and over ten realizations otherwise. Colors indicate type of experiment: Bitwise reproduction (green), qualitative reproduction (blue), altered connectivity (violet), altered plasticity mechanism (yellow). The IQR of the number of found groups for the bitwise reproduction are indicated by vertical dashed green lines; 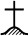 indicates algorithm failure due to too many groups (memory consumption exploded).

Obviously, it is generally not practicable to test the generality of the results with respect to every aspect of the model. However, it is certainly possible to analyze a network model to identify conceptual, parameter and numeric choices that have a high risk of being critical, and examine those with greater rigor (see our recommendations in Section 3.6.3). To demonstrate this, we pinpointed a number of such choices during the process outlined in the previous sections, and modified the qualitative reproduction developed in Section 3.3 accordingly to test them. Each modification is quite simple, and either relaxes an assumption (hidden or otherwise), shifts a parameter or alters the numerics of the dynamic components of the network model. On the basis of these generalizing reproductions of the original model, we can then determine to what extent the originally reported results are dependent on these. For all modifications, we made sure that the network dynamics are similar to the original model. The average network firing rate in the final 10 s is in the range between 2 and 8Hz (compared with 2–5Hz of the original). Raster plots, weight distributions, power spectra and parameters can be found in the Supplementary Materials. The results are summarized in Figure 8.

#### 3.4.1. Stimulus

In the original network model, the neurons are stimulated throughout the simulation by the unusual method of randomly selecting one neuron in each millisecond step, and applying a direct current of 20pA to it for the duration of that step. We replace this stimulation model with a more widely-used and biologically plausible scenario, in which each neuron receives an independent Poissonian spike train with synaptic weight of 10mV and rate of 40Hz, tuned such that the excitatory and inhibitory rates in the final second of simulation are closely matched to the original values (~ 3 spks/s).

In comparison to the original results, this scenario yields significantly different results in respect to the group statistics. Although the statistics for the longest path remain similar to the original results (data not shown), the number of found groups are reduced by around 90% to a median of 291 with an IQR of 24 (see Figure 8A *Poisson Stimulus*).

#### 3.4.2. Plasticity model

Izhikevich describes the plasticity in the original model as STDP with a time constant of τ_+_ = τ_−_ = 20 ms, *A*_+_ = 0.1mV and *A*_−_ = −0.12mV without dependence on the current strength of the synapse, i.e., of the form described by Song et al. (2000), amongst others. This form of additive STDP is known to yield bimodally distributed synaptic strengths which does not fit well to experimental observations. Clearly, an STDP rule resulting in a unimodal distribution of weights would generate qualitatively different results, but this is well known and does not need to be examined in this context. Instead, we turn our attention to several assumptions and parameters, for which biological motivation is not always easily identifiable:

1. In order to calculate the weight change the pre- and post-synaptic activity is filtered with an exponential kernel using the time constants stated above. In the default STDP synapses in NEST, the LTP/LTD traces are increased by *A*_+_/*A*_−_ leading to an “all to all” matching between pre- and post-synaptic spike pairs. The synapse model presented by Izhikevich (2006) caps the traces to a maximum value of *A*_+_/*A*_−_, leading to a “nearest neighbor” matching.
2. Synaptic weights are not updated directly after the occurrence of pre- and post-synaptic spikes. Instead, weight changes are accumulated in a separate buffer for one biological second. At the end of each simulated second, weight changes are applied to all plastic synapses simultaneously.
3. Before applying the buffered weight changes to the synaptic strengths, the buffered values are multiplied with 0.9. This reduced value is applied as an increment to the corresponding synapse and also kept as a start value for the next second. Although this mechanism lacks any biological counterpart, we refer to it as the “eligibility trace” as it introduces a very long time constant of 10s to the model. The stated intention is to have smoother development of the synaptic weights instead of the rapid and volatile development in additive STDP (Gütig et al., 2003).
4. Additionally to the weight update due to STDP, each synapse is strengthened every second by a constant value of 0.01mV. The stated motivation is to reactivate and strengthen silent neurons.

We relax these assumptions in the following ways:

1. We change the “nearest neighbor” matching to “all to all” matching. Notably, the STDP windows for a single pre/post pair look exactly the same in both cases. Interestingly, the version with “all to all” matching finds maximally 11 groups which underlines the sensitivity of the model to the exact implementation of STDP (Figure 8A *STDP window match*).
2. We vary the duration of buffering the synaptic changes in both directions. For a duration of 10 ms the simulation yields considerably more groups (median: 11, 200, IQR: 910 see Figure 8A *Buffer length* 10ms). The model also seems to be sensitive to larger buffering times, as the number of groups exploded for an increased buffer duration (10s) such that a quantitative analysis was not possible: all runs crashed due to memory limitations of our cluster (Figure 8A *Buffer length* 10s).
3. We replace the multiplication with 0.9 with an exponential decay and run the simulation for two extreme choices for the time constant: 2 s and 1,000 s, translating to a multiplicative factors of roughly 0.6 and 1.0. For the 2 s version we find 27, 000 groups in median with a high variance expressed in an IQR of 21, 600 groups (Figure 8A *Eligibility trace 2 s*).
  The 1, 000s time constants yields 13, 500 groups with an IQR of 11, 200 (Figure 8A *Eligibility trace 1,000 s*). In both cases the network is exclusively in the high gamma state. In a further experiment we disabled this eligibility trace completely. To this end, we updated the weights with the full value of the buffer after 1 s, and reset the buffered values to zero. This experiment also yields significantly more groups (10, 000) than the original model with an IQR of 2, 000 (Figure 8A *No eligibility trace*).
  We conclude that the model results are rather sensitive to this parameter, for which we can ascertain neither a plausible biological motivation nor a reason why 0.9 would be a good choice. Presumably this factor is needed to make the groups more stable over time, which is one of the main findings of the original manuscript.
4. We investigate the role of the constant additive value by setting it to zero. This seems to be completely irrelevant as the group statistics (median 2, 400 and IQR 1, 600) hardly changes with respect to the original model (Figure 8A *No additive value*). We criticize this parameter as unnecessary, introducing additional complexity to the plasticity model adding, to our understanding, no benefit.

#### 3.4.3. Connectivity

The delays in the connections are highly structured: they are evenly spread between 1 and 20ms, i.e., exactly five outgoing connections of each neuron have the delay 1ms, exactly five connections have the delay 2ms, and so on. Izhikevich (2006) argues that this very wide distribution is biologically motivated, because connections between remote neurons, that have to pass through white matter, can easily be so long. However, this is incompatible with the connection probability of 0.1, which suggests a population of neurons within the same cortical microcircuit, and thus a distribution of delays up to, at most, 2ms.

We relax the assumptions on the connectivity in two ways. First, we simulate with a uniform distribution of delays, i.e., each delay is randomly selected between 1 and 20 ms. Second, we additionally restrict the upper limit to 15, 10, and 5ms.

Unfortunately, the original group finding algorithm is not able to analyze this data as this particular delay distribution is hard wired in the C++ code, as is the integration timestep investigated in the next section. It was therefore necessary to create a more general version of this algorithm in Python, instantiating a NEST simulation, thus allowing us to perform equivalent analysis on all of our data. Due to errors in the original code which we did not re-implement in Python, our version of the algorithm finds around twice as many groups, including almost all (>99%) of the groups found by the original algorithm. A description of the Python implementation can be found in Section 2.3.2, and we provide an in-depth discussion of the errors and definition of polychrony in Section 3.5. If the original code had been designed in a flexible way allowing for potential changes to the model and its implementation, as suggested in our recommendations in Section 3.6.2, the time-consuming re-implementation of the group finding algorithm would not have been necessary.

In the experiments mentioned above, we find only a weak dependence of the group statistics on the delay distribution in the range between 10−20ms. In the cases of 20, 15, and 10ms, the simulations yield 2, 200, 900, and 7, 000 groups in median with IQRs of 700, 400, and 500 respectively (Figure 8B *delay 20 / 15 / 10 ms*). In the case of 5ms, the group statistics exhibit an extremely high median number of 35, 000 with IQR of 1, 800 (Figure 8B *delay 5 ms*). In all cases the gamma oscillations are lost, meaning (in Izhikevich's interpretation) that the network model stays “sleeping” and never “wakes up.” For the simulation with 5ms maximal delays, the network exhibits strong synchronization around 27Hz. We thus conclude that the choice of delay range beyond that found within a local cortical area is critical for the model behavior, and as such should be clearly reported.

#### 3.4.4. Neuron integration and resolution

The neuron model in the original version is integrated in 1ms steps using a form of forward Euler integration scheme:


 
  v[i]+=0.5^*^((0.04^*^v[i]+5)^*^v[i]+140-u[i]+I[i]);
  v[i]+=0.5^*^((0.04^*^v[i]+5)^*^v[i]+140-u[i]+I[i]);
  u[i]+=a[i]^*^(0.2^*^v[i]-u[i]);
 


where *v* represents the membrane potential and *u* a membrane recovery variable. This is a symplectic, or semi-implicit scheme, i.e., the update of *u* is based on an already updated value for *v*. We note several unusual features of this scheme that may result in numeric artifacts. Firstly, the forward Euler integration is a first order method, which, whilst computationally inexpensive, is less accurate than higher order methods. Secondly, the choice of 1ms as the integration time step is ten times longer than usual in computational neuroscience models, and may give inaccurate results on the single neuron level, especially in combination with the first order integration scheme. Finally, the variable v is integrated in two 0.5ms steps whilst u is integrated in one 1ms step. On the network level, forcing spikes onto a 1ms time grid may result in artefactual synchrony (Hansel et al., 1998; Morrison et al., 2007), which would in turn affect the STDP dynamics.

**Figure 9:**
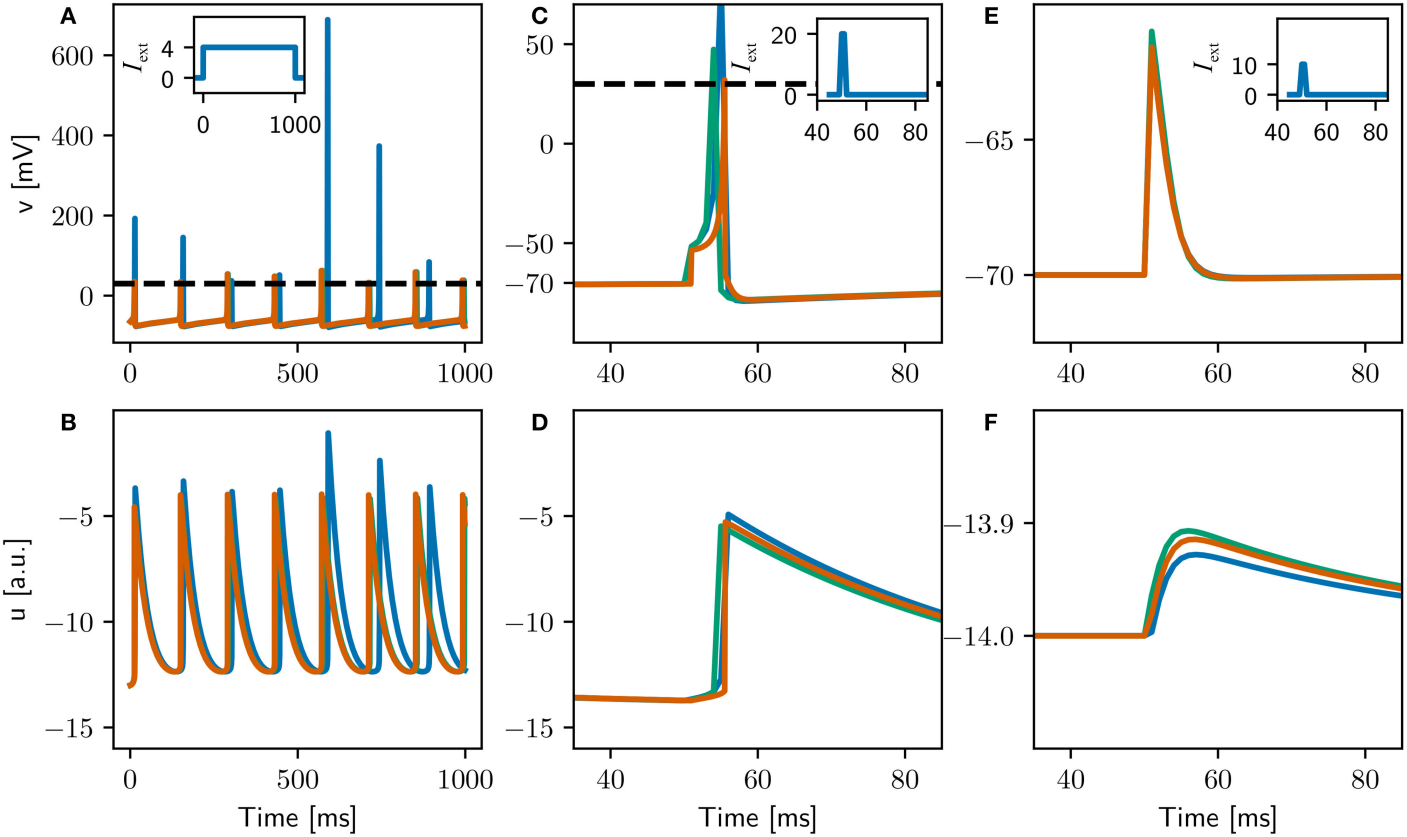
Comparison of the evolution of the membrane potential *v* (top) and the membrane recovery variable *u* (bottom) for three different configurations of the adapted Izhikevich neuron. The *original* configuration is simulated with a 1ms timestep (blue curves). The *locked* configuration performs the integration of the dynamics with a 0.1ms timestep, but spikes can only be emitted on the 1ms grid (green curves). The *high resolution* configuration is simulated with a 0.1ms timestep (orange curves). Insets depict applied currents. Dashed black line depicts action potential threshold. **(A,B)** Constant input of 4pA. **(C,D)** Two synchronous spikes of maximal weight arriving at 50ms and evoking an action potential. **(E,F)** One spike of maximal weight arriving at 50ms which does not evoke an action potential.

To consider the results of a simulation to be representative of the dynamics of the underlying model, we would expect them to show no qualitative changes if the model is re-run at a higher resolution. However, the numerical integration used in the original code is not sufficiently stable, as evidenced by the membrane voltage frequently reaching values around 1, 000mV (see Figure 9A). Consequently, simply reducing the timestep to 0.1ms may well change the single neuron dynamics, and, in turn, the network dynamics. Therefore, to investigate whether the 1ms timestep induces artefactual synchrony, we have to carefully control for all the model features that are affected by the choice of timestep.

We first adapt the neuron model to separate the numerical instability issue from the locking of spikes to a 1 ms grid, by introducing integration substeps, see also Trensch et al. (in press) for an in-depth analysis of increasing the accuracy of integration of the Izhikevich model by this method. Thus the original configuration is simulated with 1ms resolution and one integration substep: (1.0, 1). We also examine a configuration in which the numerics are integrated at a higher resolution using ten 0.1ms substeps: (1.0, 10). For this configuration, if the membrane potential crosses threshold in any substep, no further substeps are carried out in that 1ms timestep. The spike is emitted at the end of the timestep, along with the corresponding update/reset of the dynamic variables *u* and *v*. We call this the “locked” configuration, as the dynamics is integrated with high resolution but the spikes and associated neuron reset is locked to the lower resolution grid. As a comparison, we also investigate a “high resolution” configuration, in which the dynamics integration and the spike generation and reset occur on a 0.1ms grid: (0.1, 1).

As the synaptic interaction in the original is modeled as a direct current for the duration of the 1ms timestep in which it arrives, simply decreasing the timestep for the high resolution configuration would decrease the effect a spike has on the postsynaptic neuron. To adjust the synaptic weights and plasticity accordingly, we apply three criteria:

- Two synchronous incoming spikes of maximal weights elicit a post synaptic spike (as defined in Izhikevich, 2006)
- The post synaptic potential (PSP) evoked by a spike with maximal weight is conserved
- The STDP windows match

The adjusted parameters are summarized in Table 1, and the single neuron dynamics for the three configurations is illustrated in Figure 9. Unlike the original configuration, the high resolution and locked configurations exhibit a stable integration with no excessive peaks in the variables *u* and *v* when stimulated by a constant input current (Figures 9A,B). The firing rates of all three configurations are very close (see Table 1), but the locked and high resolution configurations exhibit a coefficient of variation two orders of magnitude lower than the original. The high coefficient of variation can therefore be ascribed to the numerical instability in the integration. All three configurations fit to the firing scheme of regular spiking as described in Izhikevich (2004).

**Table 1:** Comparison between the parameters, dynamics, and number of groups found for the original, locked, and high resolution neuron configurations.

	**Original**	**Locked**	**High resolution**
**PARAMETERS**			
Resolution	1.0	1.0	0.1
Integration steps	1	10	1
Delay distribution	∈[1, 20]1mssteps	∈[1, 20]1mssteps	∈[1, 20]0.1mssteps
Initial synaptic weight	6mV	6mV	50mV
Max synaptic weight	10mV	10mV	85mV
LTP	0.1	0.1	0.85
LTD	−0.12	−0.12	−1.02
Const add value	0.1mV	0.1mV	0.85mV
**SINGLE NEURON DYNAMICS (4pA CURRENT INPUT)**			
Firing rate	6.83spks/s	7.10spks/s	7.13spks/s
CV	0.124	0.004	0.003
**NETWORK DYNAMICS (LOW GAMMA)**			
Firing rate	3.28 ± 0.36spks/s	2.73 ± 0.04spks/s	2.84 ± 0.04spks/s
CV	0.39 ± 0.04	0.43 ± 0.02	0.43 ± 0.01
Fano factor (1.0ms bin)	2.21	2.29	1.89
Fano factor (0.5ms bin)	12.20	12.34	2.91
Spectral peak	≈40Hz	≈27Hz	≈25Hz
**GROUPS FOUND**			
	4, 300 ± 2, 900	13, 000 ± 1, 100	151 ± 25

The responses of the three configurations to spiking input (Figures 9C–F) indicate that the first two criteria stated above have been fulfilled (data not shown for third criterion), indicating that the three configurations can be meaningfully compared in a full network simulation. Note that the curves for the locked and high resolution configurations are still distinguishable, because the high resolution configuration can emit spikes on a 0.1ms grid whereas the locked configuration can only emit them on a 1ms grid.

To remove synchronization artifacts in the full network simulation of the high resolution configuration due to the distribution of delays in multiples of 1 ms, we draw the delays for the excitatory-excitatory connections from a uniform distribution between 1.0 and 20.0ms with a resolution of 0.1ms. To allow the fairest comparison, for all configurations we use the original input stimulus (one neuron made to fire randomly selected to fire each millisecond by current injection of an input current of twice maximal synaptic weight), as we previously showed in Section 3.4.1 that a Poissonian stimulus reduces the number of groups found by around 90%. The network activity for the full network simulations of the locked and high resolution exhibits average firing rates that are very close to each other and slightly lower than the original; the coefficients of variation are comparable across all three configurations (around 3spks/s and 0.4, respectively, see Table 1). The spectral peak is found at around 40Hz for the original, but around 25Hz for the locked and high resolution configurations. We thus conclude that the gamma band oscillation is an artifact of the low resolution of the integration step. In terms of synchrony, the Fano factor measured with a binsize of 1.0ms yields slightly higher values for the original and locked configurations (2.21, 2.29) than for the high resolution configuration (1.89). However, with a bin size of 0.5ms the synchrony induced by the 1.0ms spike locking is clearly visible. The original and locked configurations have a much increased Fano factor of around 12, whereas the high resolution network simulation increases only slightly to around 3.

Applying our Python reproduction of the polychronous group finding algorithm to the network results of all three configurations yields 4,300 (IQR 2,900) groups for the original, 13, 000 (IQR 1,100) groups for the locked, and 151 (IQR 25) groups for the high resolution configuration. These results are indicated by the labels *Improved integration* and *Resolution* 0.1ms in Figure 8B.

Thus, in summary, the key difference between the original and the locked configuration is that the latter integrates the dynamics without the numerical instabilities of the former. Resolving this issue causes an increase in the number of groups by a factor of three. The difference between the locked and the high resolution simulation is that spikes and delays occur on a 0.1ms grid rather than a 1ms grid. This decreases the number of groups found by a factor of 90 (and by a factor of 30 from the original).

We therefore conclude that the number of groups found is strongly influenced by the choice of a 1ms timestep and delay resolution, although the network dynamics, in terms of firing rate and coefficient of variation, is not. In particular, the original study significantly overstates the number of groups to be found in such networks, due to the artificial synchrony induced by these implementational (rather than conceptional) choices. Using standard numerics or testing the robustness of the results for a higher simulation precision as recommended in Section 3.6.3 could have prevented this misinterpretation in the original study.

### 3.5. Definition of polychrony

In section 3.4, we examined the sensitivity of polychronous group generation to parameter settings and model assumptions, given comparable network dynamics. We now turn our attention to the group finding algorithm itself. As stated in the previous section, it was necessary to re-implement the original analysis script in order to investigate the effects of alternative choices of delay distribution and integration timestep.

In this process we found several aspects of the original algorithm, briefly outlined in section 2.3.2, that warrant further discussion and investigation. First, we note that the identification of a polychronous group is based on an analysis of the connectivity, rather than the activity. The original manuscript reports that ~ 90% of the groups which are found to be stable over 24 h of simulation time can also be found to be active in the spiking activity. However, this part of the analysis is not part of the available online materials, and so we were not able to confirm this relationship.

Second, we found three major errors in the C++ implementation of the algorithm:

- During the simulation phase, the spike delivery buffer often overflows, leading to a spike being delivered at the wrong time. Although this mainly happens in large groups, we consider this to be a critical error as exact spike timings are necessary to reliably activate groups.
- A group is only valid if the maximum layer, the longest chain of neurons within the group, is greater than, or equal to seven. However, the calculation of layers depends on an arbitrary sorting of neuron ids and also on the time of activation of those neurons. This leads to errors in which neurons are assigned to the wrong layer. If this results in a sub-threshold number of layers, the group will be considered invalid and not counted.
- The maximal duration of the simulation is set to 1ms after the last spike delivery, however two simultaneous spike arrivals lead to a postsynaptic spike generation of up to 4ms later. This last spike is thus overlooked in the original algorithm. This is a crucial error, as missing the last spike can result in a reduced number of layers identified for a group, and therefore to the group being considered invalid (i.e., less than seven layers).

In our re-implementation of the algorithm we fixed these errors; as a result, our algorithm finds around twice as many groups, but including more than 99% of those found by the original algorithm.

Thirdly, we note that the motivation for many of the conditions underlying the definition of a polychronous group is unclear; for example, the exclusion of weak synapses from the analysis, or the classification of groups that are activated by only one or two neurons as invalid. In particular, the analysis algorithm sets the seemingly arbitrary conditions that a polychronous group has to consist of at least six neurons and seven layers. The choice of the number of layers has a profound effect on the number of groups found. Reducing it to five increases the number of groups found in the original model from 4, 305 to 27, 116, whereas increasing it to ten decreases the number to 608. As no scientific justification is given for the choice of seven, we speculate that it was chosen for aesthetic reasons. In any case, the strong dependence of the results on the choice of thresholding parameter indicate that it should be explicitly stated as a model critical parameter, even though it is not a parameter of the network model.

**Figure 10:**
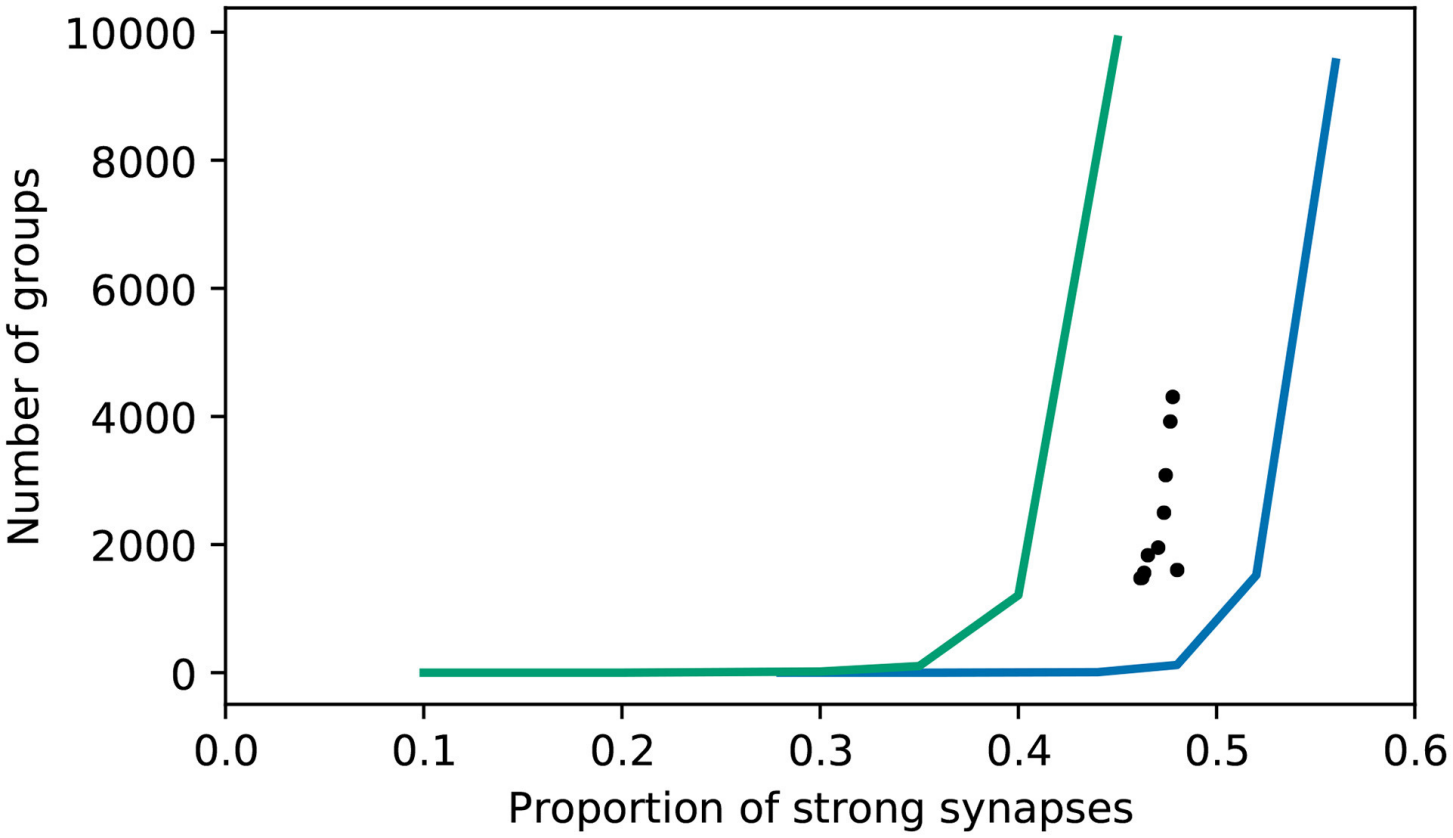
Number of groups identified by the original algorithm as a function of the proportion of excitatory synapses that are strong. Realizations of the bitwise identical reproduction; black dots. Surrogate data with connections randomly selected to be strong (excitatory-excitatory synapses only—all excitatory-inhibitory synapses are strong); blue curve. Surrogate data with connections randomly selected to be strong (all excitatory); green curve.

To get an understanding of how many groups are found with respect to those expected from a network with random connectivity, we performed a surrogate analysis. The original C++ code provides a similar functionality, by shuffling the excitatory-excitatory connections. However, it is not clear why the number of groups found with this shuffling defines the null hypothesis, given that the excitatory-inhibitory connections also adapt during the course of the simulation (and almost all become strong). Regarding the strength of these as a given introduces a bias. Moreover, the functionality does not allow the proportion of strong synapses to be considered as an independent variable. We therefore developed a surrogate analysis in which excitatory connections (either just excitatory-excitatory, or all excitatory) are randomly drawn with a given probability of being strong. The results are shown in Figure 10.

In line with the original findings, networks with randomly selected strong excitatory-excitatory synapses exhibit fewer groups (using the original group finding algorithm) than those where the strong synapses develop due to network activity. The proportion of random strong synapses must be increased to around ~ 50% in order to find as many groups as in the “grown” networks, where the proportion of strong synapses is around ~ 45%. Note that in this setting, 20% of synapses are automatically strong, being the excitatory-inhibitory connections. However, if the strong synapses are randomly selected from all excitatory synapses, the opposite tendency is found: only around ~ 40% strong synapses are required for the group finding algorithm to identify as many groups as in the grown network. Hence, the algorithm finds either more or fewer groups in grown networks than random networks, depending on what assumption is used to generate the latter.

We therefore conclude that the provided analysis script is an additional factor undermining the reproducibility of the original study. It contains coding errors that distort the results, making it likely that a researcher trying to re-implement the analysis would generate substantially different numbers of groups, even if the network model had been reproduced identically. These errors could have been avoided, or at least made more visible, by clean code features such as encapsulation and commenting, as discussed in Section 3.2.2 and summarized in Section 3.6.2. Moreover, there is an unstated strong dependence on an apparently arbitrary threshold parameter, and the null hypothesis from which positive results are to be distinguished is not well motivated. For future research into polychronous groups, we would therefore suggest following a different analytic approach. Some alternative methods (none of which were available at the time of publication of the original study) are discussed in Section 4.3.

### 3.6. Recommendations

In carrying out the steps outlined in sections 2.2–3.5, we identified which features of the code and the methodology of the original study support reproducing the results, and which hinder it. From these we derive a series of recommendations that, if followed, would not only increase the reproducibility of a given study, but also its scientific credibility, by reducing the risk that results are dependent on implementational details.

These can be roughly divided into three categories, although of course there is overlap. On the most basic level, it is important to make the code available and executable. This includes topics such as sharing and providing an installation guide, as well as information about the versions used of model code and any dependencies. On the next level, we provide recommendations on how to make it comprehensible and testable. This covers topics from low-level artifacts like commenting and naming of parameters and functions, to more abstract issues such as appropriate organization of the code and unit tests for components. Finally, our work revealed that computational models may easily contain undesirable implementation dependencies. Whereas it is not feasible to comprehensively test for all of them, we emphasize the importance of using existing standards as much as possible, both for accuracy and comprehensibility. In addition, we uncovered an alternative dynamical mode of the original network, with a lower peak in the power spectrum, which occurs in a minority of simulation runs. This illustrates the importance of carrying out multiple runs of models to uncover any dependency on the random seed used.

#### 3.6.1. Make code available and executable

##### Recommendation: share the code

We would certainly not have been able to reproduce the simulation results either identically or qualitatively if the author had not provided the complete source. This applies not only to the network model but also to the analysis of the results. As there are many options for sharing the code in a sustainable fashion, as listed below, “available from the author on request” should not be considered an adequate fulfillment of this recommendation. Moreover, the code should be accessible for the reviewers when an article is submitted to a journal, and not deferred until publication, so that the reviewers can form an opinion of its reproducibility.

*Modeldb*. ModelDB^3^ is a database for computational and conceptual models in neuroscience. One can choose to share the code on ModelDB itself or as a weblink to the code. ModelDB provides a direct link between publication and source code of the model. Additionally, the database can be searched by keywords, for example for specific neuron models, types of plasticity or brain regions. Since a model can even be entered into ModelDB as a link to another hosting platform, there is really no reason not to make an entry.

*Zenodo*. Zenodo^4^ makes it possible to assign a DOI to a certain version of the code. The code version will also be archived on the CERN cloud infrastructure. The model code can be stored in a github repository and then linked and archived via Zenodo.

*Github, gitlab, bitbucket*. Web-based hosting services such as GitHub, GitLab, and Bitbucket^5^,^6^,^7^ are mainly based on git, a standard and widely used tool in collaborative software development. The advantage of sharing model code through git is that it facilitates opening up the code to the community.

*Open source brain*. The Open Source Brain platform is a web resource for publishing and sharing models in the field of computational neuroscience with a strong focus on open source technologies. The submitted models can be visualized and their parameter spaces and dynamics can be explored in browser-based simulations (Gleeson et al., 2018).

*Collaboratory*. The collaboratory is a web portal designed within the Human Brain Project intended to improve the quality of collaboration between many possibly international parties (Senk et al., 2017). It allows scientists to share data, collaborate on code and re-use models and methods, and enables tracking and crediting researchers for their contributions.

##### Recommendation: provide an installation guide

The single stand-alone C++ program downloaded for this study was easy to install. However, more complicated set-ups with dependencies on other applications (e.g., simulation or analysis tools) require more work. An installation guide takes (most of) the guesswork out of it. An installation guide should not only include the exact steps and commands to install the software, but should also name the platform and operating system on which the authors tested the installation steps. Additionally, it should mention a complete list of software dependencies.

##### Recommendation: use a version control system

In the current investigation, the downloaded script did not match the paper, and the C++ and MATLAB versions did not match each other. Therefore it was not clear which version of the code had been used to generate the reported results. More generally, models are often developed further after being published, which leads to increasing divergence between the description in the manuscript and the current version of the model. A version control system such as git, SVN or Mercurial helps to keep different versions accessible, and enables users and scientists to understand the changes to the implementation of a model.

##### Recommendation: provide provenance tracking

To reproduce the study, we used specific versions of NEST and various Python packages. However, sometimes different versions of software applications vary significantly in their performance (Gronenschild et al., 2012) or just have non-compatible APIs. The manuscript and the installation guide should be specific about which versions of software were used to generate the results.

#### 3.6.2. Make code comprehensible and testable

##### Recommendation: modularize the code

Separating simulation and data analysis makes it possible to use the two independently. In this study, it allowed us to apply a new analysis to the original simulation results and, vice versa, the original analysis code to our implementation of the model. Without the possibility to apply exactly the same analysis to different implementations, we would not have been able to discover the causes of the disparities between the original network model and our initial attempts to reproduce it.

##### Recommendation: encapsulate the code

Encapsulation gathers data and the methods that operate on it into cohesive units. This is a similar principle to modularization. Using methods with meaningful names rather than operating directly on data makes the code easier to comprehend; compare:


I[i]+=s[firings[k][1]][delays[firings[k][1]][t-firings[k][0][j]];


and


deliver_spike(post_neuron, weight(pre_neuron, post_neuron))


Further, it makes the code less error prone, as complex access/operation routines are defined in one place and parameterized, rather than repeated throughout the code. Finally, it facilitates testing, see below.

##### Recommendation: write flexible code

Code flexibility is a precondition for efficient testing of model robustness toward changes on both the implementation level (e.g., smaller integration time step) and the modeling level (e.g., different parameter values). Testing the generalized reproducibility of the model (see Section 3.4) was a tedious and time-consuming process due to several model features being hardwired into the simulation and analysis code. Routines should be written as general as possible to avoid these problems; using standard tools (see next section) will tend to mitigate this issue automatically.

##### Recommendation: provide tests

Reproducing the synapse model was challenging, because there were discrepancies between our initial model and the original and handling specific combinations of pre- and post-synaptic spikes that were not defined in the original publication. To avoid this situation, novel network elements such as neuron, synapse or stimulus models should be accompanied by tests that define the output of the model for representative or critical inputs. This documents the behavior of the model, especially for border cases, in much greater detail than it would be reasonable to include in a text description.

##### Recommendation: comment the code

Using comments substantially increases the comprehensibility of the code, and thus the ability of a researcher to re-implement it in a different framework. Comments should explain *what* complex code sections are doing, but often straightforward code sections are greatly enhanced by a comment explaining *why* they are performing their operations.

##### Recommendation: parameterize meaningfully and consistently

Parameters should be given meaningful names such that they can be understood when they occur in an expression. Parameter definitions should be gathered in parameter files, or at the beginning of a stand-alone script, rather than spread throughout. Raw numbers (other than 0 and 1) should not appear in expressions, as this reduces the comprehensibility of the code, and they are easy to overlook as parameters that influence the behavior of the model.

##### Recommendation: use parameter files

Models often rely on a set of parameters which should be either declared in the beginning of the source code, or, if there are more than a few parameters, in a separate file. To aid comprehensibility, if there is more than one experiment conducted with one model there should be a dedicated parameter file for each experiment, with a corresponding human-readable table as proposed by Nordlie et al. (2009).

##### Recommendation: use tables to communicate parameters

It is easy to overlook a parameter when writing a text description of a network model. Use of structured tables, such as those proposed by Nordlie et al. (2009) acts as a reminder to record all the model parameters and their values, and present them in a comprehensible and easily referable fashion for the reader.

#### 3.6.3. Reduce risk of implementation dependencies

##### Recommendation: use standard tools

Tools that are created and maintained by a group of developers over a period of time and have a substantial user base will generally have more consistently applied coding standards, documentation and tests than a homebrewed single-purpose application. All these aspects increase the comprehensibility of the code and reduce the risk that it contains numeric or algorithmic errors. Given the current excellent availability of open source tools for simulation and data analysis, using standard tools should be preferred over homebrew as far as possible. Novel network elements (e.g., neuron model) or analyses should ideally be handled by contributing features to open source tools, or at least formating them as compatible patches. The use of homebrewed simulation or analysis tools should be clearly motivated, and such code should comply with the recommendations set out here.

##### Recommendation: use standard numerics

Using standard numerics lowers the risk of introducing rounding or other numeric errors and also makes it easier to understand the code.

##### Recommendation: perform multiple realizations

A robust model will generate statistically equivalent results for different choices of the random seed. Variable behavior should be reported; this is not only relevant for a reader's ability to evaluate the explanatory power of the model, it is also important for reproducibility to be aware that the model can yield substantially different results.

##### Recommendation: test model robustness

Proofs of model robustness with regard to implementation details, parameter values, and higher-level modeling choices boost the quality and credibility of the presented scientific results. A basic requirement of model robustness is that an outcome of a simulation that is reported as model behavior should not change qualitatively if the simulation is repeated with higher precision. In case of the model that we investigate here, increasing the simulation resolution significantly affects the number of polychronous groups found (see Section 3.4.4), and hence, renders the main result of the study questionable. Such checks should always be carried out if there is a risk that the results might be distorted by artificial synchrony or numeric instabilities.

## 4. Discussion

In this article, we have demonstrated that even if model code is available and can be executed on a local machine with only minimal modifications (Section 3.1), this is only a first step toward enabling reproduction of a study. By taking the publicly available model code of a well-known study (Izhikevich, 2006) and attempting to reproduce it in NEST, we uncovered a variety of barriers to reproducibility (sections 3.2, 3.3). From each of these barriers, we derive a recommendation that would lower or remove it.

These recommendations are explained in detail in the previous section, and for convenience we have gathered them into a checklist, which is available in the Supplementary Material. This checklist can be used by researchers to evaluate and improve the reproducibility of their neuronal network model before submitting an article. Similarly, a reviewer can use it to rapidly assess the likely reproducibility of a submitted model, without having to expend considerable time trying to actually reproduce it.

Beyond the practical steps that can be taken to improve the quality of model code and related artifacts, in the course of our study we have identified several unusual assumptions and numerics choices in the original simulation and analysis code, and investigated to what extent the reported model behavior depends on them (sections 3.4, 3.5).

With regards to the model code, in the case of the implementation of background noise (random selection in each millisecond of a neuron to fire), the non-standard features of the plasticity model, and the extremely long range of delays, making a choice that was better biologically founded (or at least removed complexity that did not have a clear biological foundation) resulted in a reduction or increase in the number of groups found by an order of magnitude. In the case of the simulation resolution, despite careful matching of network and single neuron dynamics, in a network running at a higher resolution of 0.1ms, we found a massive reduction of groups compared to either the original network, or one with 0.1ms integration but spikes locked to a 1.0ms grid. This last finding is of particular concern, as it demonstrates that the majority of the polychronous groups reported in the original study can be attributed to artificial synchrony brought about by an unsuitable choice of numerics (low resolution).

Similarly, with regards to the analysis code, we discovered a series of coding errors that distorted the findings, and strong dependencies on both a thresholding parameter (lacking a biological motivation) and the assumptions defining the null hypothesis.

Thus we conclude that the main reported results of the original study generalize very poorly. The number of groups found varies substantially with each aspect we investigated, with the exception of the additive factor in the plasticity model, which seems to have no effect. We argue that had the dependence of the findings on a very specific configuration of modeling and implementation choices been apparent, the original study would not have been as influential as it has been.

Clearly, it is not possible to check for all parameter and implementation choices, and it is reasonable to assume that the authors of the current study have more computational resources at their disposal for such analyses than were available to the author of the original. This notwithstanding, we note that it is the obligation of authors to evaluate their choices and assumptions critically, and to be transparent about which ones are necessary for the reported results. Analogously, it is the obligation of reviewers to use their expertise to identify potential dependencies and request additional simulations to uncover them.

As discussed in the next section, following the set of recommendations laid out in Section 3.6 would not only increase the ability of researchers to independently verify the findings of neuronal network studies, but would decrease the likelihood that such findings are subject to highly specific parameter and implementation choices.

### 4.1. Relationship of the reproducibility guideline to scientific quality

The reproducibility guideline is divided into three categories. The first category contains recommendations that allow researchers to reproduce identical results, which includes also the case where a researcher wants to rerun a simulation at a later point in time. The second category of recommendations facilitate qualitative reproduction by others, primarily through effective communication of the model code and parameters. The recommendations of the third category principally address model robustness. All three categories are important for the quality and credibility of the presented scientific results, but on different levels. By following the recommendations of the first category, a researcher can be transparent about exactly what experiments were carried out using which software. Following the second category provides evidence to other researchers that the study was conducted in a structured way. Moreover, a study that follows these recommendations invites other researchers to investigate the model independently. A study that follows the third category of recommendations demonstrates the researchers' ability to critically assess their own work, their willingness to disclose limitations, and their openness to potential refutation of the results by other researchers in future studies.

### 4.2. Limitations of the reproducibility guideline

The reproducibility guideline developed in the course of this study is not intended as a definitive document, and the authors welcome suggestions for further recommendations to increase the currently poor record of reproducibility in neuronal network modeling studies. In particular, our guideline is aimed at the reproducibility of networks of point (or few-compartment) neuron models. Where as much of it is likely applicable to networks of biophysical neuron models with thousands of compartments (commenting code is never a bad idea), some recommendations are likely to be inappropriate (e.g., using tables to communicate parameters) and some important aspects that boost reproducibility may well have been overlooked entirely. The adaptation of the guideline to such models lies outside the expertise of the authors. We suspect that domain specific languages such as NEUROML (Gleeson et al., 2018) have an important role to play here, as they provide unambiguous, standardized, and machine-readable representations of complex neurons and their connectivity.

### 4.3. Alternative methods for detecting polychronous groups

Izhikevich (2006) introduces a method to find polychonous groups in the connectivity data of the presented spiking neural network model. Although the concept is fruitful in this very specific case, it does not generalize to other means. More general methods (e.g., Torre et al., 2013; Quaglio et al., 2017; Russo and Durstewitz, 2017) have recently been developed for the detection of repeated precise spike sequences in electrophysiological recordings. Such methods do not use any assumption of the underlying connectivity and could be applied to the simulated spiking activity in order to find active patterns without the prior detection of potential polychronous groups in the connection profile. With these methods the same kind of analysis could be performed as in Izhikevich (2006) with the advantageous possibility of comparing the results to experimental data in order to confirm the validity of the model.

## 5. Conclusion and outlook

Based on our work to reproduce the network presented by Izhikevich (2006), we conclude that the more points in the guideline are adhered to, the easier it will be to reproduce a study of a network of spiking neurons, and the higher quality the study will be. Whereas journals are beginning to take issues such as availability of model code more seriously than before, the current study clearly demonstrates that this is a necessary but not sufficient condition for reproducibility. We propose that the editorial boards of journals in computational neuroscience go considerably further, and provide their reviewers with clearly defined reproducibility criteria, for which we provide a draft. Only in this way can we achieve a substantial change in attitude and approach in our field.

## Author contributions

SK and AM created a prototype of the project. RP created all figures. RP, PW, and AM investigated and eliminated the discrepancies between the original code and the NEST model. RP and PW performed the analysis and simulations. SK, PW, and RP created the NEST group finding algorithm. All authors jointly wrote the manuscript.

### Conflict of interest statement

The authors declare that the research was conducted in the absence of any commercial or financial relationships that could be construed as a potential conflict of interest.

## Footnotes

^1^https://github.com/INM-6/reproducing-polychronization

^2^https://www.izhikevich.org/publications/spnet.htm

^3^https://senselab.med.yale.edu/modeldb/

^4^https://zenodo.org/

^5^https://github.com/

^6^https://gitlab.com/

^7^https://bitbucket.org
