# Supplementary material for: Reproducing Polychronization: A Guide to Maximizing the Reproducibility of Spiking Network Models
Source: Front Neuroinform. 2018 Aug 3;12:46. doi: 10.3389/fninf.2018.00046 (PMC6085985; doi:10.3389/fninf.2018.00046)
Supplement: Supplementary file 1 [file Data_Sheet_1.pdf]

Checklist for Authors and Reviewers to
Maximize Reproducibility of Spiking Neural Network Models

Make code available and executable	
☐	Share the model of your code on a model hosting site<br>(e.g. ModelDB, Open SourceBrain)<br>or a general purpose service (e.g. GitHub, Bitbucket)
☐	Provide an installation guide for all dependencies<br>and the model itself
☐	Use a version control system and tag the used version
☐	Provide provenance tracking<br>to explicitly declare versions of all used software

Make code comprehensible and testable	
☐	Modularize the code
☐	Encapsulate the code
☐	Write flexible code
☐	Apply unit tests
☐	Comment the code with natural language
☐	Use meaningful parameter names
☐	Use parameter files containing all model parameters
☐	Use tables to document all model parameters

Reduce risk of implementation dependencies	
☐	Use standard tools wherever possible
☐	Use standard numerics
☐	Perform multiple realizations
☐	Test model robustness

Version 1.0
26.06.2018

1 Supplementary Material

1.1 Changes to the original C++ code

To reproduce the results in the manuscript, we used the C++ implementation of the model poly_spnet.cpp from Izhikevichs website (http://www.izhikevich.org/publications/spnet.htm). Unfortunately, the C++ code can not be compiled with the standard g++ compiler under Ubuntu 16.04 LTS.

Following adaptations were made to compile the code:

```
– line 1: #include <iostream.h> –> #$include <iostream>
– change all occurrences of 'cout' to 'std::cout'
– line 764: void main() –> int main()
```

After successful compilation we had to fix a few runtime errors:

```
– Comment out line 955
– Comment out line 957
```

After these changes the simulation and analysis ran through but did not save the data about polychronous groups. To change this:

```
– Comment in line 691 to 697
```

To avoid the computation with shuffled data (and possibly overwriting data):

```
– Comment out line 957 to 960
```

2 Network Description

1 shows the parameter for the network model.

A	Model summary	
Populations	Two	
Topology	None	
Connectivity	Population specific	
Neuron model	Izhikevich	
Channel models	None	
Synapse model	δ current input	
Plasticity	Izhikevich STDP	
Measurements	Spike activity	
**B**	**Populations**	
**Name**	**Elements**	**Size**
Exc	Izhikevich	800
Inh	Izhikevich	200
**C**	**Connectivity**	
**Source**	**Target**	**Pattern**
Exc	Exc + Inh	Random, fixed-outdegree 100, plastic, initial weight 6
Inh	Exc	Random fixed-outdegree 100, static, weight -5
**D**	**Neuron and synapse model**	
Type	Izhikevich	
Dynamics	$v = v + h0.5(0.04V + 5)v + 140 - u + I + I_{syn}$	
	$v = v + h0.5(0.04V + 5)v + 140 - u + I + I_{syn}$	
	$u = u + ha(0.2vu)$	

Table 1: Description of the network model (according to Nordlie 2009).

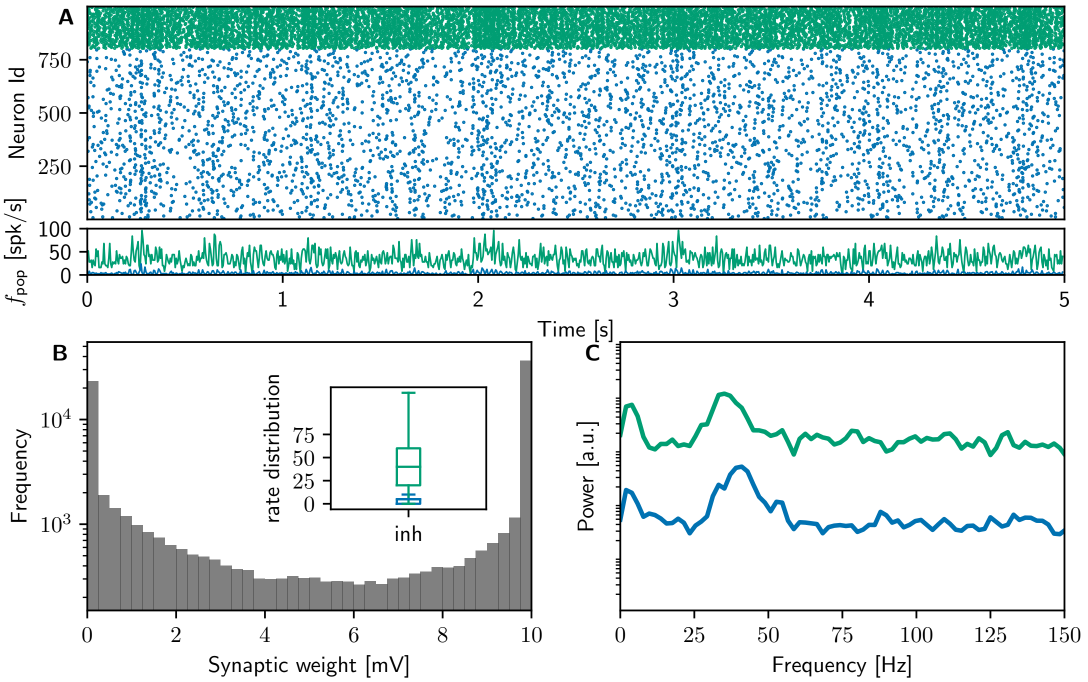

A	simulation-params
rec_spikes	10000.0
resolution	1.0
sim-time	18000000.0
rec_mem	0.0
synapse-update-interval	1000
neuron-integration-steps	1
data-path	data/NEST_model/

B	stimulus
rate	40.0
distribution	poisson
type	generate
weight	10.0

C	plasticity
W_inh	-5.0
synapse-model	stdp_izh_synapse
Wmax	10.0
W_init	6.0
LTP	0.1
tau_syn_update_interval	10000.0
reset_weight_change_after_update	False
constant_additive_value	0.01
LTD	-0.12

D	connectivity
type	generate
delay-distribution	uniform-non-random
delay-range	[1, 21]

E	initial-state
type	generate
distribution	uniform
V_m-range	[-65, -55]

Parameters for experiment poisson stimulus

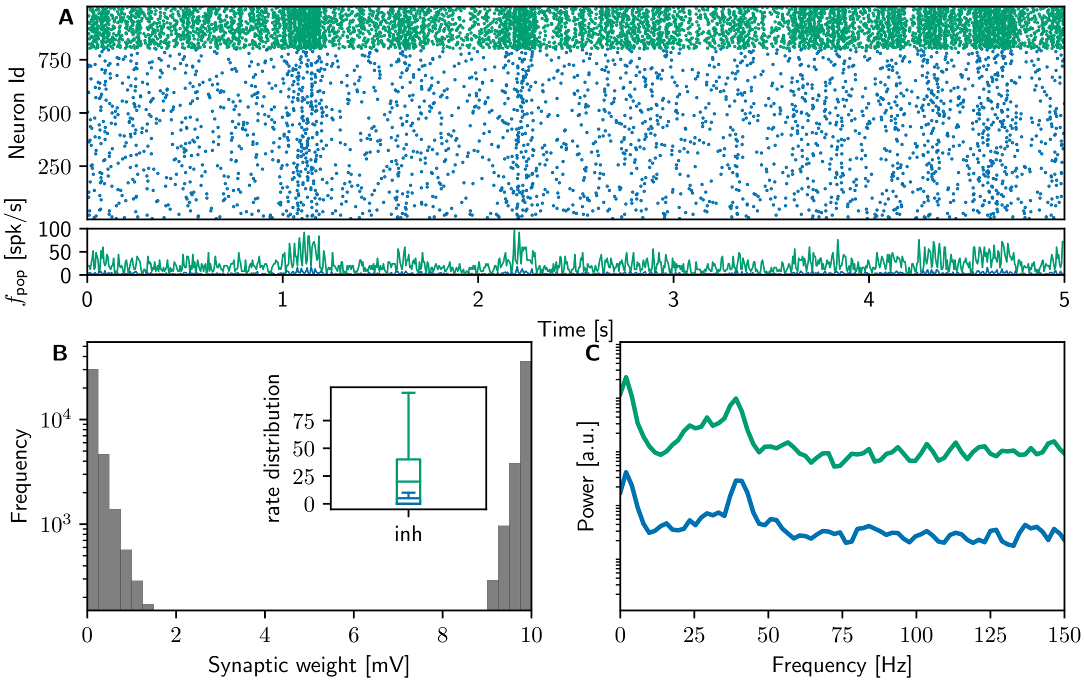

A	simulation-params
rec_spikes	10000.0
resolution	1.0
sim-time	18000000.0
rec_mem	0.0
synapse-update-interval	1000
neuron-integration-steps	1
data-path	data/NEST_model/
**B**	**stimulus**
distribution	original
type	generate
**C**	**plasticity**
W_inh	-5.0
synapse-model	stdp_izh_synapse
Wmax	10.0
W_init	6.0
LTP	0.1
tau_syn_update_interval	0.0
reset_weight_change_after_update	True
constant_additive_value	0.0
LTD	-0.12
**D**	**connectivity**
type	generate
delay-distribution	uniform-non-random
delay-range	[1, 21]
**E**	**initial-state**
type	generate
distribution	uniform
V_m-range	[-65, -55]

Parameters for experiment time driven additive 1s

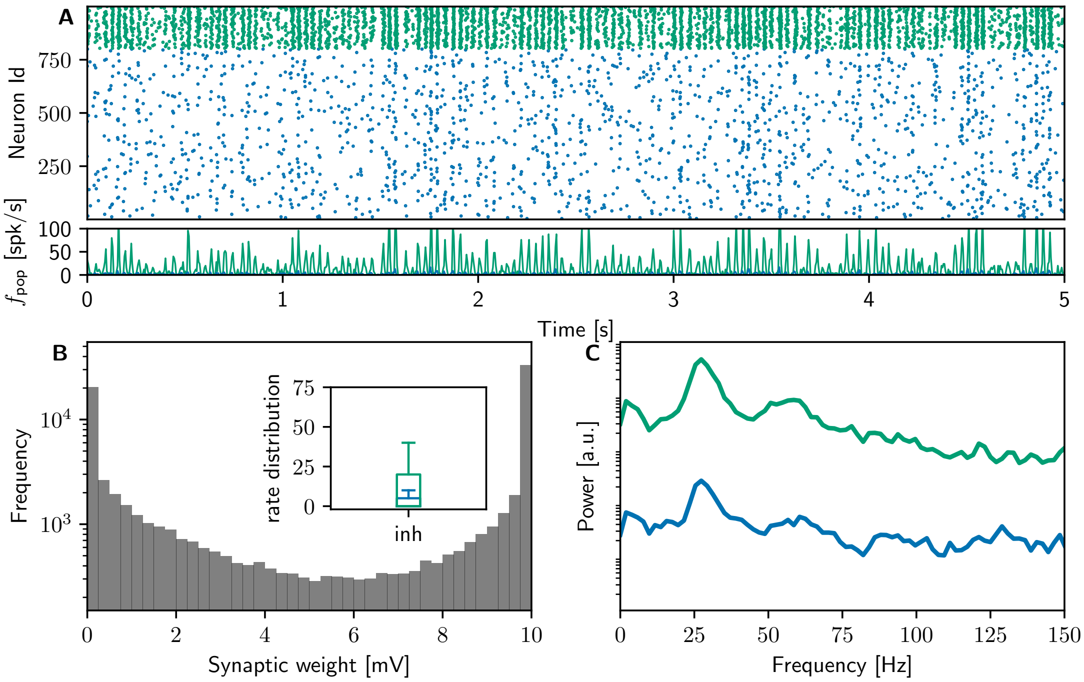

A	simulation-params
rec_spikes	10000.0
resolution	1.0
sim-time	18000000.0
rec_mem	0.0
synapse-update-interval	1000
neuron-integration-steps	1
data-path	data/NEST_model/
**B**	**stimulus**
distribution	original
type	generate
**C**	**plasticity**
W_inh	-5.0
synapse-model	stdp_izh_synapse
Wmax	10.0
W_init	6.0
LTP	0.1
tau_syn_update_interval	10000.0
reset_weight_change_after_update	False
constant_additive_value	0.01
LTD	-0.12
**D**	**connectivity**
type	generate
delay-distribution	uniform-random
delay-range	[1, 6]
**E**	**initial-state**
type	generate
distribution	uniform
V_m-range	[-65, -55]

Parameters for experiment delay distribution 5

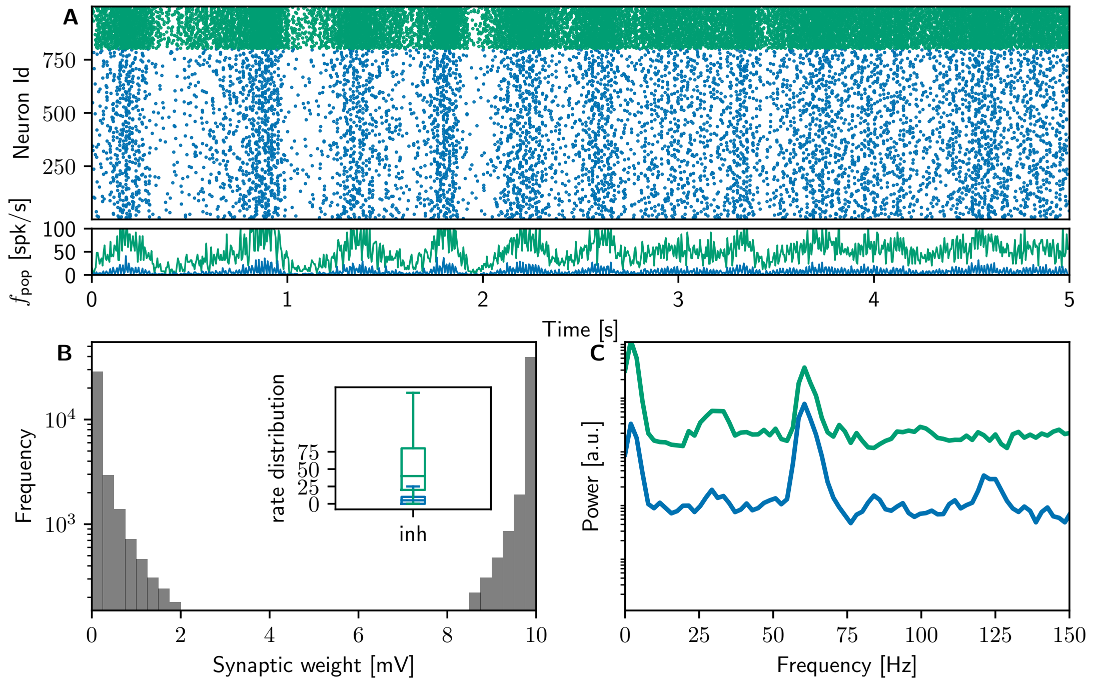

A	simulation-params
rec_spikes	10000.0
resolution	1.0
sim-time	18000000.0
rec_mem	0.0
synapse-update-interval	1000
neuron-integration-steps	1
data-path	data/NEST_model/
**B**	**stimulus**
distribution	original
type	generate
**C**	**plasticity**
W_inh	-5.0
synapse-model	stdp_izh_synapse
Wmax	10.0
W_init	6.0
LTP	0.1
tau_syn_update_interval	2000.0
reset_weight_change_after_update	False
constant_additive_value	0.01
LTD	-0.12
**D**	**connectivity**
type	generate
delay-distribution	uniform-non-random
delay-range	[1, 21]
**E**	**initial-state**
type	generate
distribution	uniform
V_m-range	[-65, -55]

Parameters for experiment tau syn update interval 2s

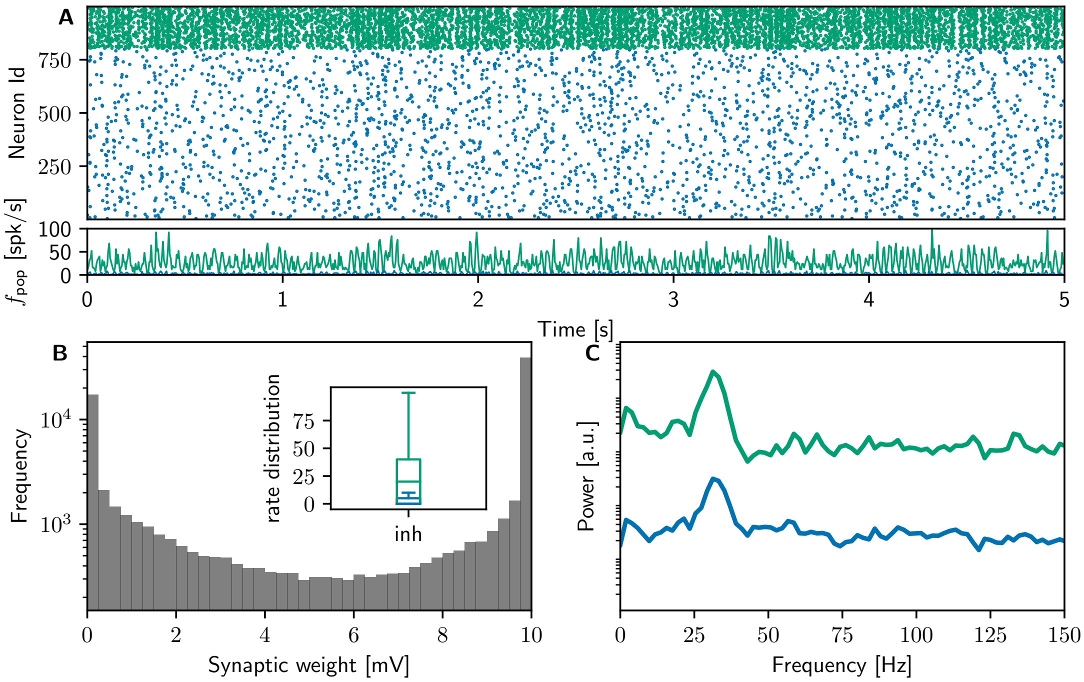

A	simulation-params
rec_spikes	10000.0
resolution	1.0
sim-time	18000000.0
rec_mem	0.0
synapse-update-interval	1000
neuron-integration-steps	10
data-path	data/NEST_model/

B	stimulus
distribution	original
type	generate

C	plasticity
W_inh	-5.0
synapse-model	stdp_izh_synapse
Wmax	10.0
W_init	6.0
LTP	0.1
tau_syn_update_interval	10000.0
reset_weight_change_after_update	False
constant_additive_value	0.01
LTD	-0.12

D	connectivity
type	generate
delay-distribution	uniform-non-random
delay-range	[1, 21]

E	initial-state
type	generate
distribution	uniform
V_m-range	[-65, -55]

Parameters for experiment qualitative model high res

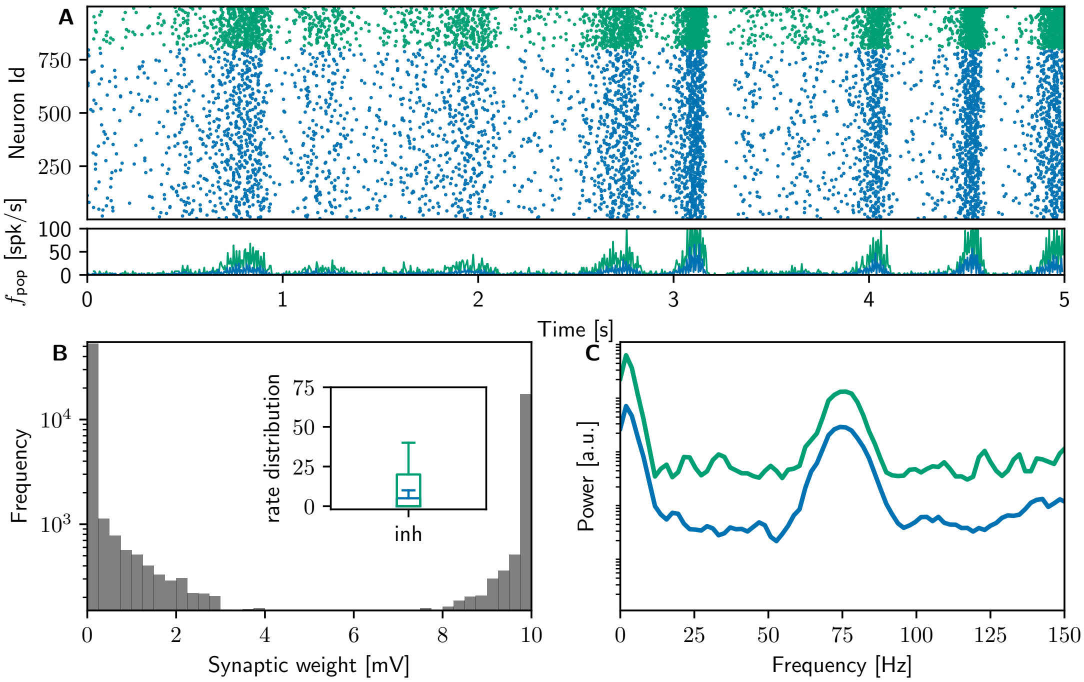

A	simulation-params
rec_spikes	10000.0
resolution	1.0
sim-time	18000000.0
rec_mem	0.0
synapse-update-interval	1000
neuron-integration-steps	1
data-path	data/NEST_model/
**B**	**stimulus**
type	reproduce
from-file	data/original_model/bitwise_reproduction/{rep}/stim.dat
**C**	**plasticity**
W_inh	-5.0
synapse-model	stdp_izh_new_naive_synapse
Wmax	10.0
W_init	6.0
LTP	0.1
LTD	-0.12
**D**	**connectivity**
type	reproduce
from-file	data/original_model/bitwise_reproduction/{rep}/connectivity.json
**E**	**initial-state**
type	reproduce
from-file	data/original_model/bitwise_reproduction/{rep}/vuinit.dat

Parameters for experiment stdp window match

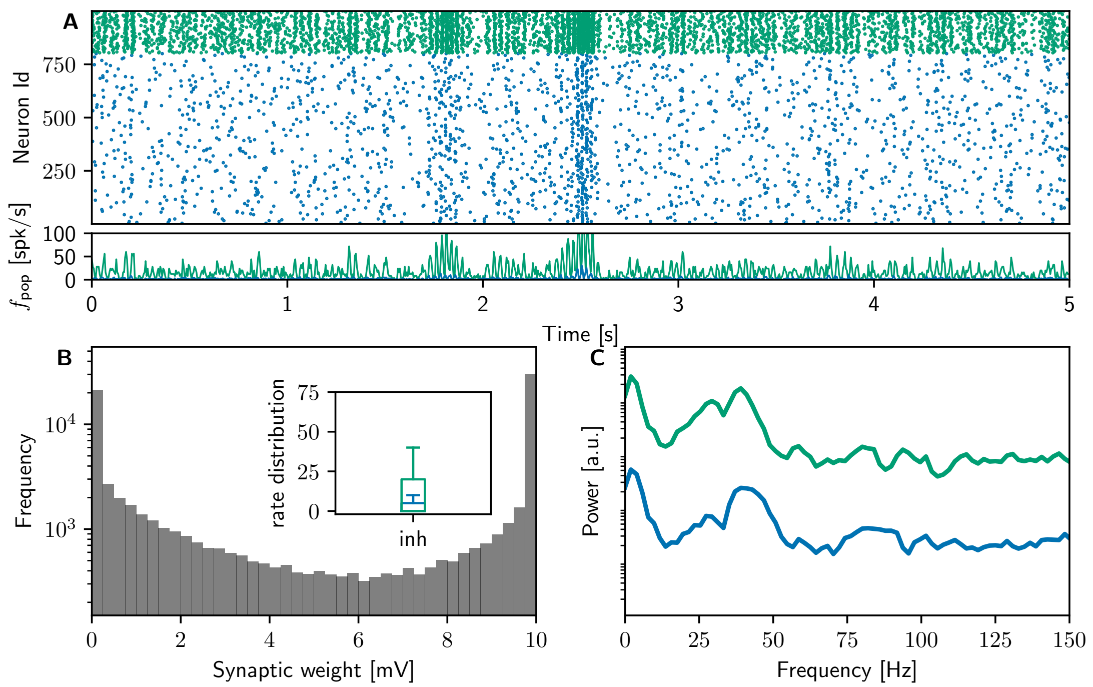

A	simulation-params
rec_spikes	10000.0
resolution	1.0
sim-time	18000000.0
rec_mem	0.0
synapse-update-interval	1000
neuron-integration-steps	1
data-path	data/NEST_model/
**B**	**stimulus**
distribution	original
type	generate
**C**	**plasticity**
W_inh	-5.0
synapse-model	stdp_izh_synapse
Wmax	10.0
W_init	6.0
LTP	0.1
tau_syn_update_interval	10000.0
reset_weight_change_after_update	False
constant_additive_value	0.01
LTD	-0.12
**D**	**connectivity**
type	generate
delay-distribution	uniform-random
delay-range	[1, 16]
**E**	**initial-state**
type	generate
distribution	uniform
V_m-range	[-65, -55]

Parameters for experiment delay distribution 15

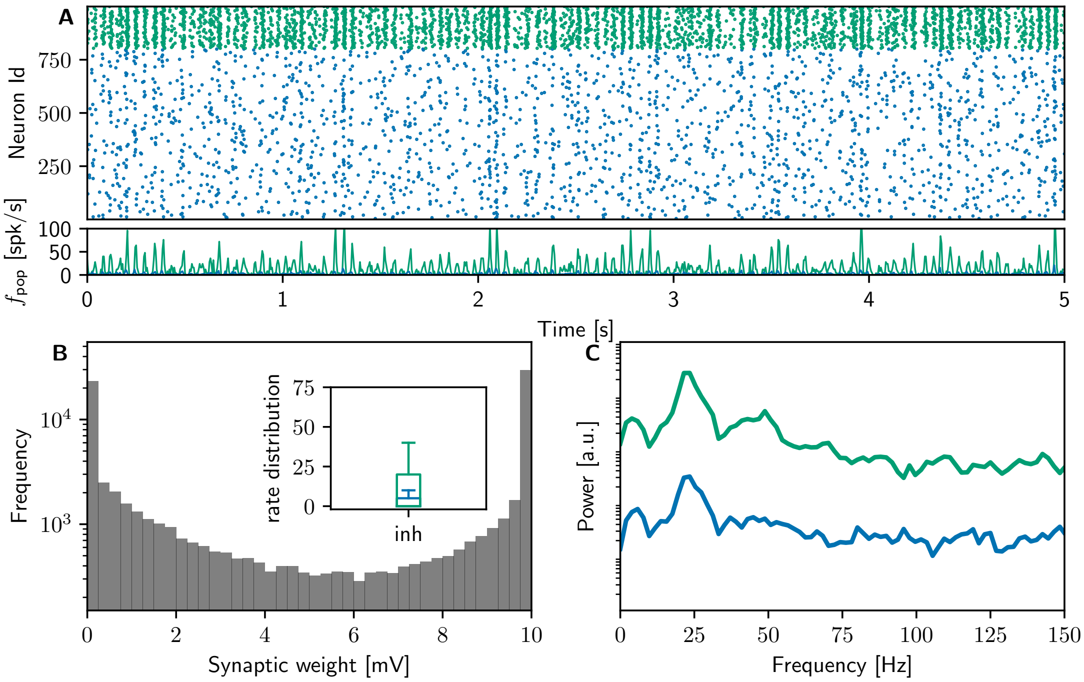

A	simulation-params
rec_spikes	10000.0
resolution	1.0
sim-time	18000000.0
rec_mem	0.0
synapse-update-interval	1000
neuron-integration-steps	1
data-path	data/NEST_model/
**B**	**stimulus**
distribution	original
type	generate
**C**	**plasticity**
W_inh	-5.0
synapse-model	stdp_izh_synapse
Wmax	10.0
W_init	6.0
LTP	0.1
tau_syn_update_interval	10000.0
reset_weight_change_after_update	False
constant_additive_value	0.01
LTD	-0.12
**D**	**connectivity**
type	generate
delay-distribution	uniform-random
delay-range	[1, 11]
**E**	**initial-state**
type	generate
distribution	uniform
V_m-range	[-65, -55]

Parameters for experiment delay distribution 10

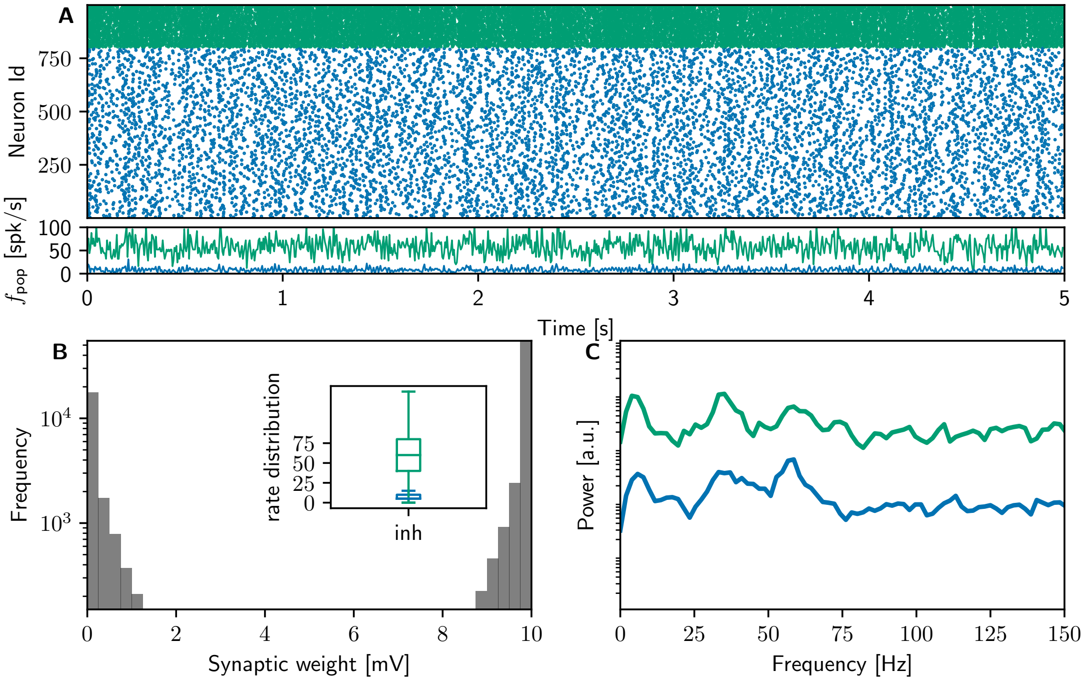

A	simulation-params
rec_spikes	10000.0
resolution	1.0
sim-time	18000000.0
rec_mem	0.0
synapse-update-interval	10000
neuron-integration-steps	1
data-path	data/NEST_model/
**B**	**stimulus**
distribution	original
type	generate
**C**	**plasticity**
W_inh	-5.0
synapse-model	stdp_izh_synapse
Wmax	10.0
W_init	6.0
LTP	0.1
tau_syn_update_interval	10000.0
reset_weight_change_after_update	False
constant_additive_value	0.01
LTD	-0.12
**D**	**connectivity**
type	generate
delay-distribution	uniform-non-random
delay-range	[1, 21]
**E**	**initial-state**
type	generate
distribution	uniform
V_m-range	[-65, -55]

Parameters for experiment synapse update interval 10s

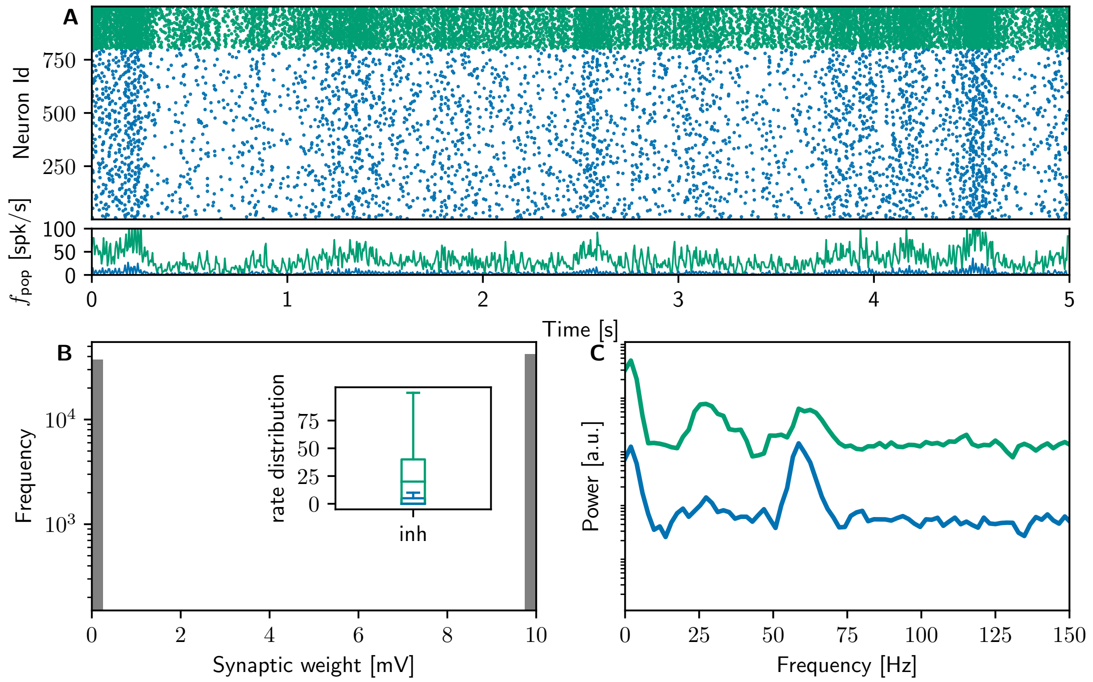

A	simulation-params
rec_spikes	10000.0
resolution	1.0
sim-time	18000000.0
rec_mem	0.0
synapse-update-interval	1000
neuron-integration-steps	1
data-path	data/NEST_model/
**B**	**stimulus**
distribution	original
type	generate
**C**	**plasticity**
W_inh	-5.0
synapse-model	stdp_izh_synapse
Wmax	10.0
W_init	6.0
LTP	0.1
tau_syn_update_interval	100000.0
reset_weight_change_after_update	False
constant_additive_value	0.01
LTD	-0.12
**D**	**connectivity**
type	generate
delay-distribution	uniform-non-random
delay-range	[1, 21]
**E**	**initial-state**
type	generate
distribution	uniform
V_m-range	[-65, -55]

Parameters for experiment tau syn update interval 1000s

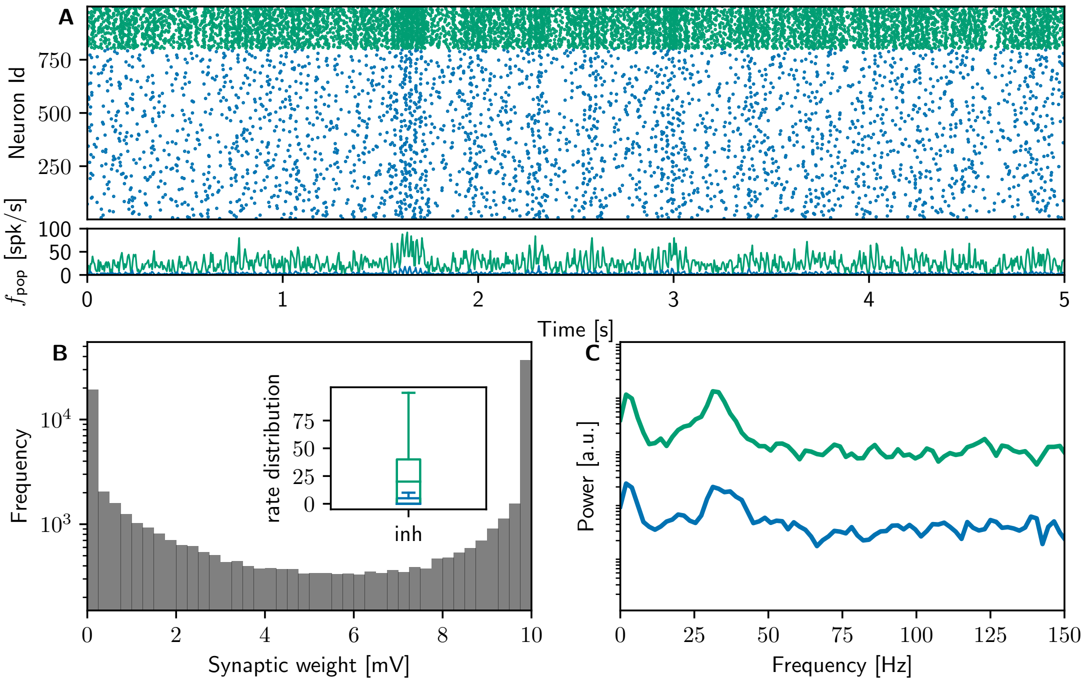

A	simulation-params
rec_spikes	10000.0
resolution	1.0
sim-time	18000000.0
rec_mem	0.0
synapse-update-interval	1000
neuron-integration-steps	1
data-path	data/NEST_model/
**B**	**stimulus**
distribution	original
type	generate
**C**	**plasticity**
W_inh	-5.0
synapse-model	stdp_izh_synapse
Wmax	10.0
W_init	6.0
LTP	0.1
tau_syn_update_interval	10000.0
reset_weight_change_after_update	False
constant_additive_value	0.01
LTD	-0.12
**D**	**connectivity**
type	generate
delay-distribution	uniform-random
delay-range	[1, 21]
**E**	**initial-state**
type	generate
distribution	uniform
V_m-range	[-65, -55]

Parameters for experiment delay distribution 20

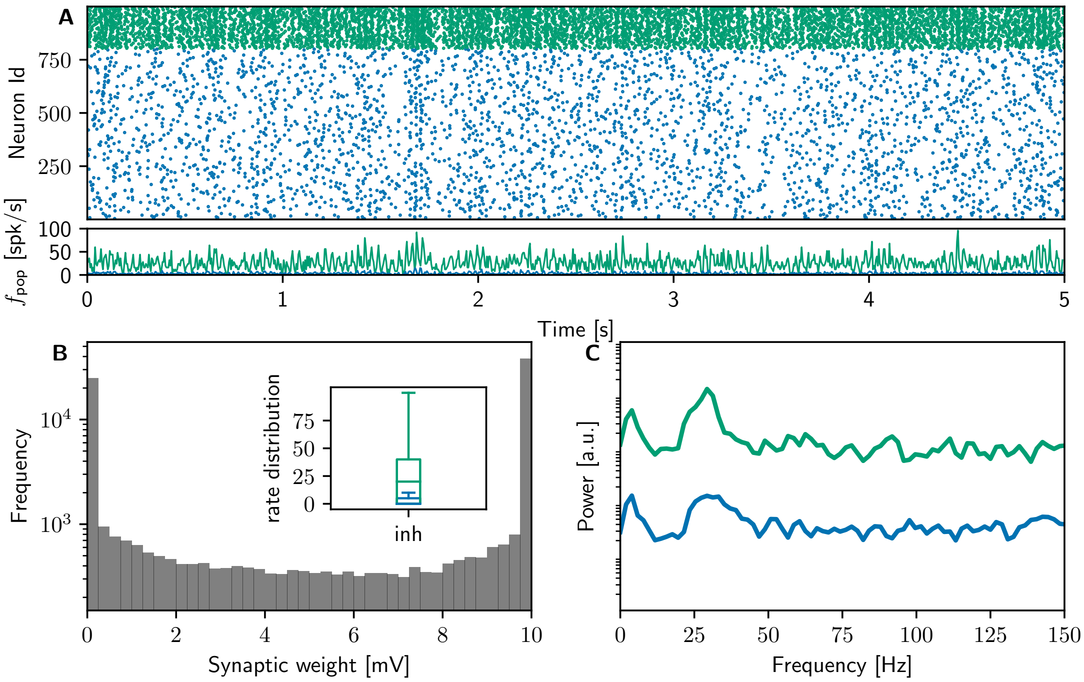

A	simulation-params
rec_spikes	10000.0
resolution	1.0
sim-time	18000000.0
rec_mem	0.0
synapse-update-interval	100
neuron-integration-steps	1
data-path	data/NEST_model/
**B**	**stimulus**
distribution	original
type	generate
**C**	**plasticity**
W_inh	-5.0
synapse-model	stdp_izh_synapse
Wmax	10.0
W_init	6.0
LTP	0.1
tau_syn_update_interval	10000.0
reset_weight_change_after_update	False
constant_additive_value	0.01
LTD	-0.12
**D**	**connectivity**
type	generate
delay-distribution	uniform-non-random
delay-range	[1, 21]
**E**	**initial-state**
type	generate
distribution	uniform
V_m-range	[-65, -55]

Parameters for experiment synapse update interval 0p1s

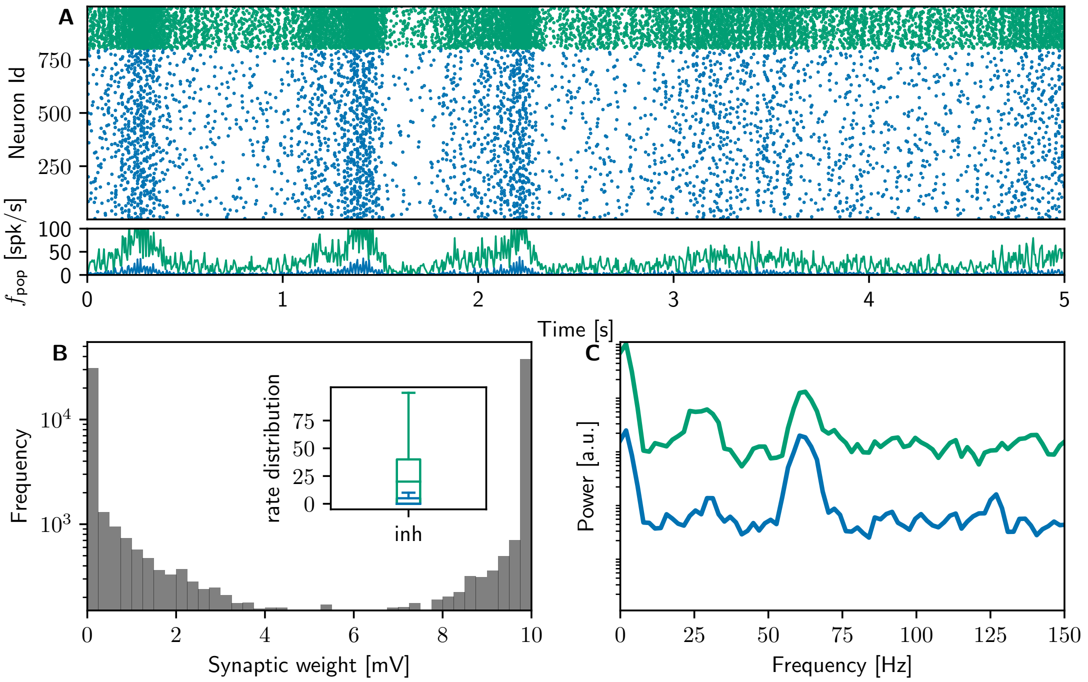

A	simulation-params
rec_spikes	10000.0
resolution	1.0
sim-time	18000000.0
rec_mem	2000.0
synapse-update-interval	1000
neuron-integration-steps	1
data-path	data/NEST_model/
**B**	**stimulus**
type	reproduce
from-file	data/original_model/bitwise_reproduction/{rep}/stim.dat
**C**	**plasticity**
W_inh	-5.0
synapse-model	stdp_izh_bitwise_correct_synapse
Wmax	10.0
W_init	6.0
LTP	0.1
LTD	-0.12
**D**	**connectivity**
type	reproduce
from-file	data/original_model/bitwise_reproduction/{rep}/connectivity.json
**E**	**initial-state**
type	reproduce
from-file	data/original_model/bitwise_reproduction/{rep}/vuinit.dat

Parameters for experiment bitwise reproduction

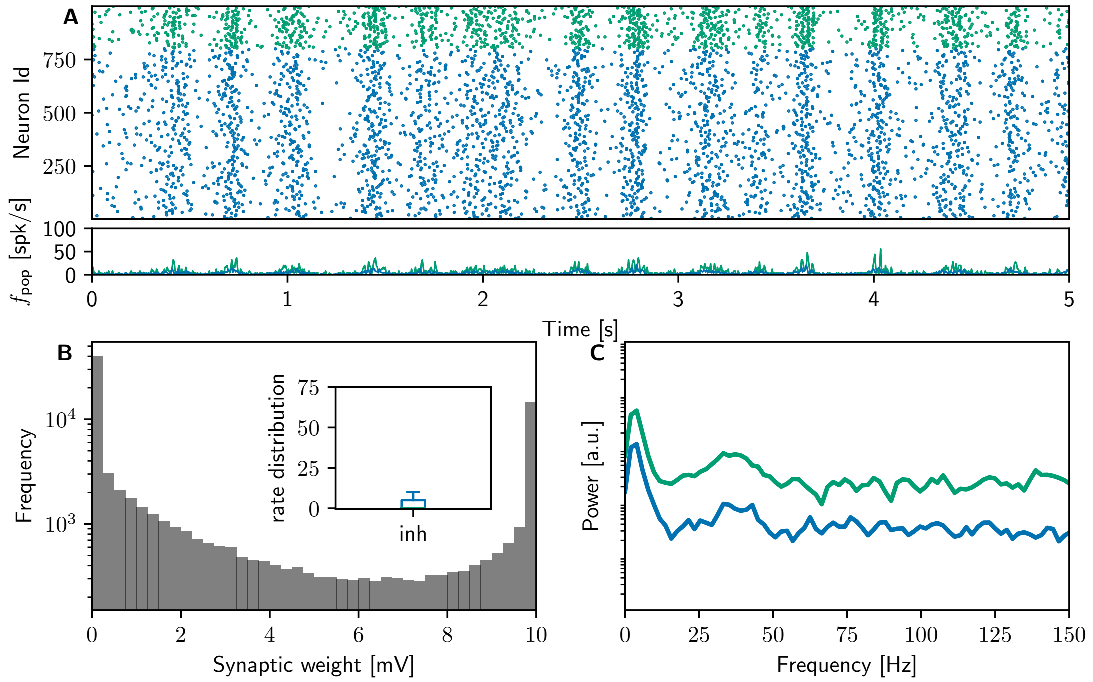

A	simulation-params
rec_spikes	10000.0
resolution	1.0
sim-time	18000000.0
rec_mem	0.0
synapse-update-interval	1000
neuron-integration-steps	1
data-path	data/NEST_model/
**B**	**stimulus**
type	reproduce
from-file	data/original_model/bitwise_reproduction/{rep}/stim.dat
**C**	**plasticity**
W_inh	-5.0
synapse-model	stdp_izh_naive_synapse
Wmax	10.0
W_init	6.0
LTP	0.1
LTD	-0.12
**D**	**connectivity**
type	reproduce
from-file	data/original_model/bitwise_reproduction/{rep}/connectivity.json
**E**	**initial-state**
type	reproduce
from-file	data/original_model/bitwise_reproduction/{rep}/vuinit.dat

Parameters for experiment initial reproduction

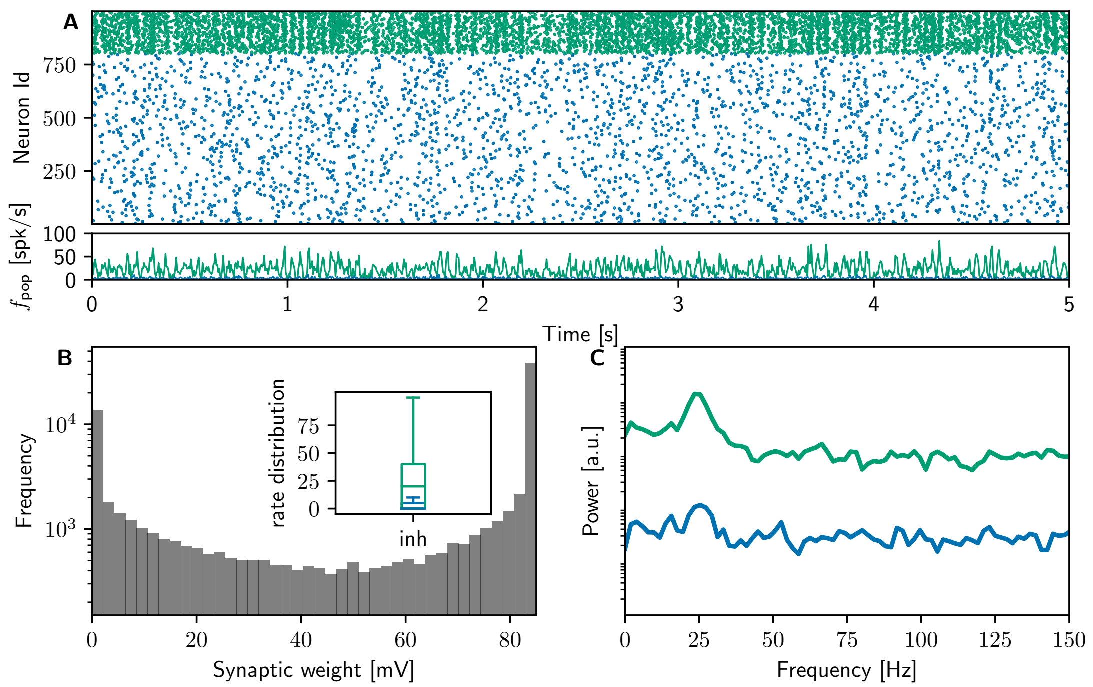

A	simulation-params
rec_spikes	10000.0
resolution	0.1
sim-time	18000000.0
rec_mem	1000.0
synapse-update-interval	1000
neuron-integration-steps	1
data-path	data/NEST_model/
**B**	**stimulus**
distribution	original
type	generate
**C**	**plasticity**
W_inh	-35.0
synapse-model	stdp_izh_synapse
Wmax	85.0
W_init	50.0
LTP	0.85
tau_syn_update_interval	10000.0
reset_weight_change_after_update	False
constant_additive_value	0.085
LTD	-1.02
**D**	**connectivity**
type	generate
delay-distribution	uniform-random
delay-range	[1, 21]
**E**	**initial-state**
type	generate
distribution	uniform
V_m-range	[-65, -55]

Parameters for experiment resolution 0p1 W pspmatched

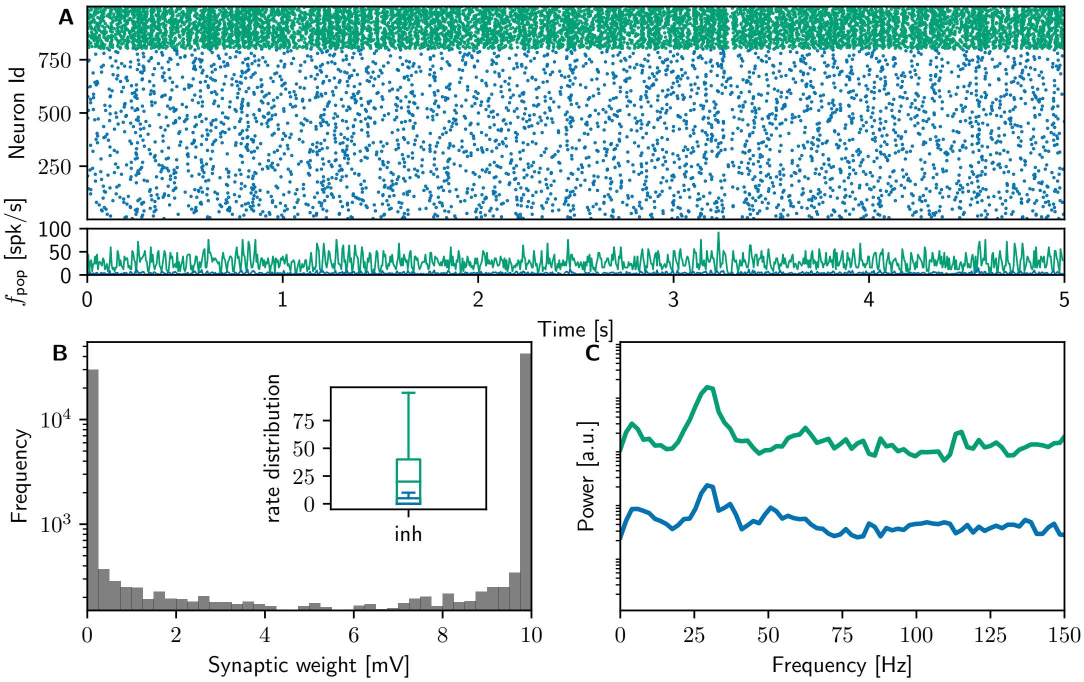

A	simulation-params
rec_spikes	10000.0
resolution	1.0
sim-time	18000000.0
rec_mem	0.0
synapse-update-interval	10
neuron-integration-steps	1
data-path	data/NEST_model/
**B**	**stimulus**
distribution	original
type	generate
**C**	**plasticity**
W_inh	-5.0
synapse-model	stdp_izh_synapse
Wmax	10.0
W_init	6.0
LTP	0.1
tau_syn_update_interval	10000.0
reset_weight_change_after_update	False
constant_additive_value	0.01
LTD	-0.12
**D**	**connectivity**
type	generate
delay-distribution	uniform-non-random
delay-range	[1, 21]
**E**	**initial-state**
type	generate
distribution	uniform
V_m-range	[-65, -55]

Parameters for experiment synapse update interval 0p01s

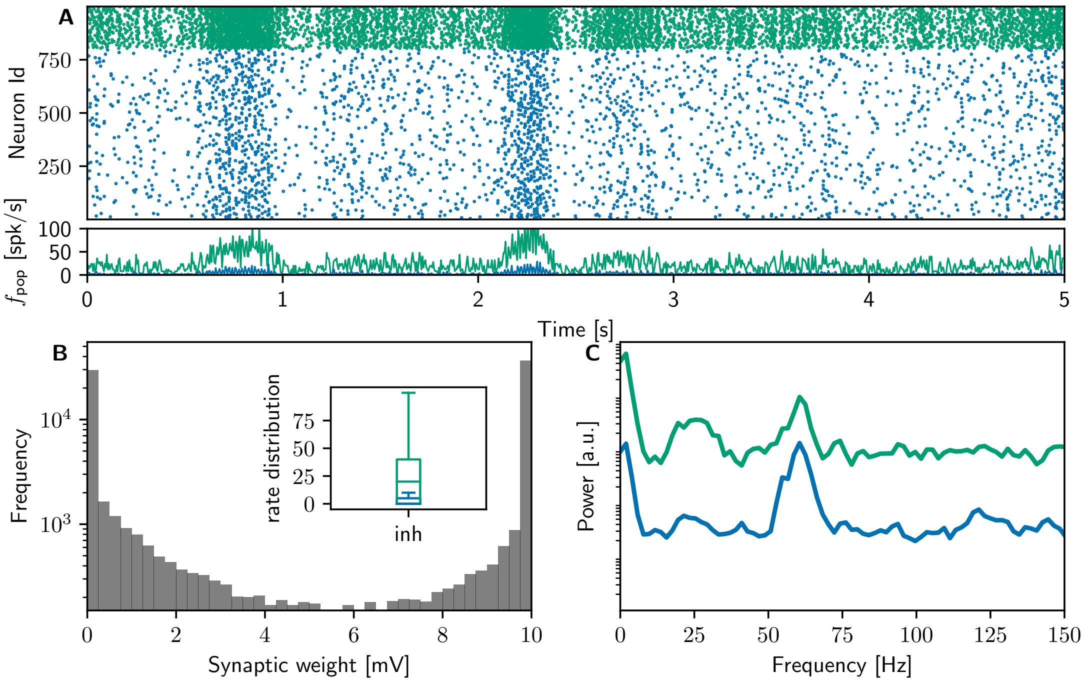

A	simulation-params
rec_spikes	10000.0
resolution	1.0
sim-time	18000000.0
rec_mem	0.0
synapse-update-interval	1000
neuron-integration-steps	1
data-path	data/NEST_model/
**B**	**stimulus**
distribution	original
type	generate
**C**	**plasticity**
W_inh	-5.0
synapse-model	stdp_izh_synapse
Wmax	10.0
W_init	6.0
LTP	0.1
tau_syn_update_interval	10000.0
reset_weight_change_after_update	False
constant_additive_value	0.0
LTD	-0.12
**D**	**connectivity**
type	generate
delay-distribution	uniform-non-random
delay-range	[1, 21]
**E**	**initial-state**
type	generate
distribution	uniform
V_m-range	[-65, -55]

Parameters for experiment const add value 0p0

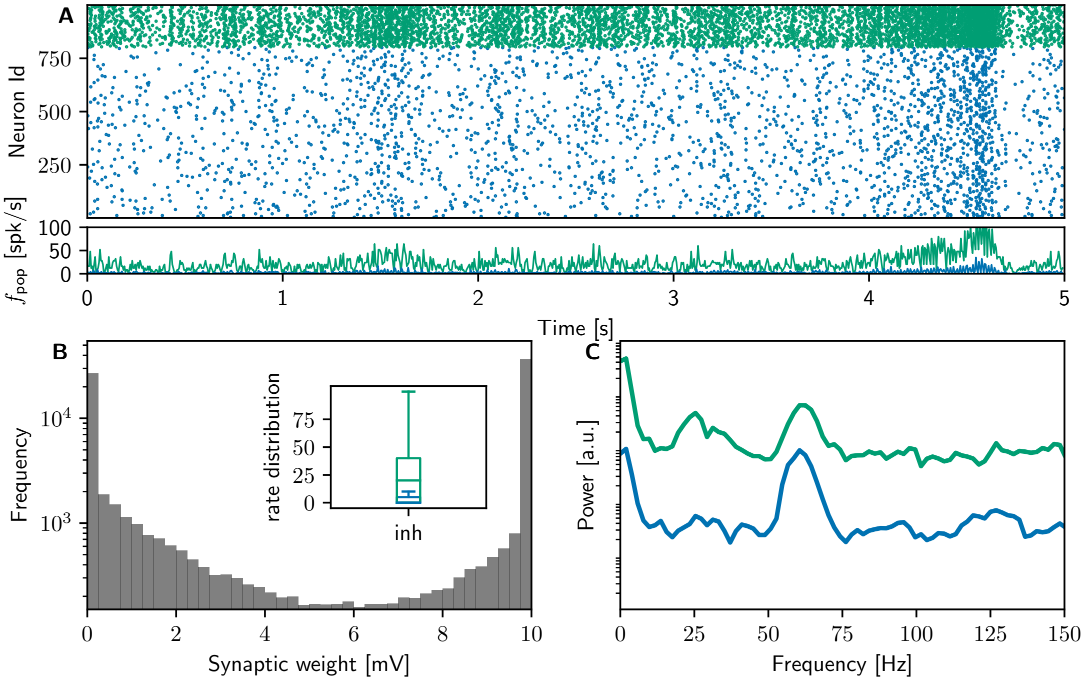

A	simulation-params
rec_spikes	10000.0
resolution	1.0
sim-time	18000000.0
rec_mem	0.0
synapse-update-interval	1000
neuron-integration-steps	1
data-path	data/NEST_model/
**B**	**stimulus**
distribution	original
type	generate
**C**	**plasticity**
W_inh	-5.0
synapse-model	stdp_izh_synapse
Wmax	10.0
W_init	6.0
LTP	0.1
tau_syn_update_interval	10000.0
reset_weight_change_after_update	False
constant_additive_value	0.01
LTD	-0.12
**D**	**connectivity**
type	generate
delay-distribution	uniform-non-random
delay-range	[1, 21]
**E**	**initial-state**
type	generate
distribution	uniform
V_m-range	[-65, -55]

Parameters for experiment qualitative model
